# Supplementary figures and images for: Bactericidal type IV secretion system homeostasis in Xanthomonas citri
Source: PLoS Pathog. 2020 May 26;16(5):e1008561. doi: 10.1371/journal.ppat.1008561 (PMC7286519; doi:10.1371/journal.ppat.1008561)

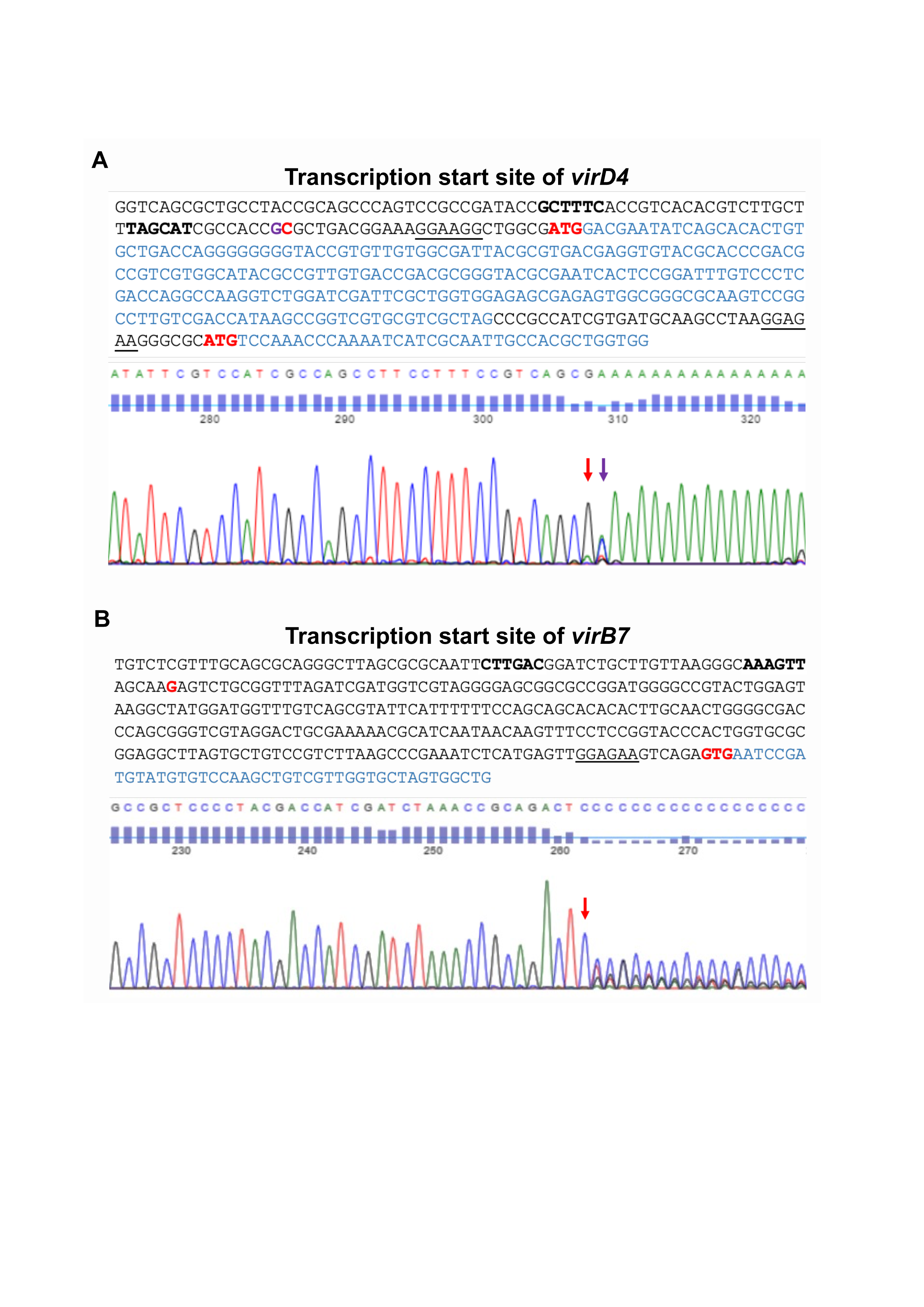

Supplement: S1 Fig — A) Bottom panel shows the two adjacent possible transcription start sites (TSS) of virD4 (red and purple arrow) due to sequencing ambiguity. The possible start sites are indicated with a purple G and a red C in the nucleotide sequence (top panel). The virD4 sequence starts at the end of the displayed nucleotide sequence. In between the TSS and virD4 a putative open reading frame (ORF) coding for a protein of unknown function can be found. B) Similar analysis for the virB7 with TSS depicted with red arrow (bottom panel) and red G in the nucleotide sequence (top panel). In parts A and B, translation start sites are depicted in red and open reading frames in blue. Putative RNA polymerase binding sites are shown in bold and putative ribosome binding sites are underlined in regular font. (TIF) [file ppat.1008561.s001.tif]

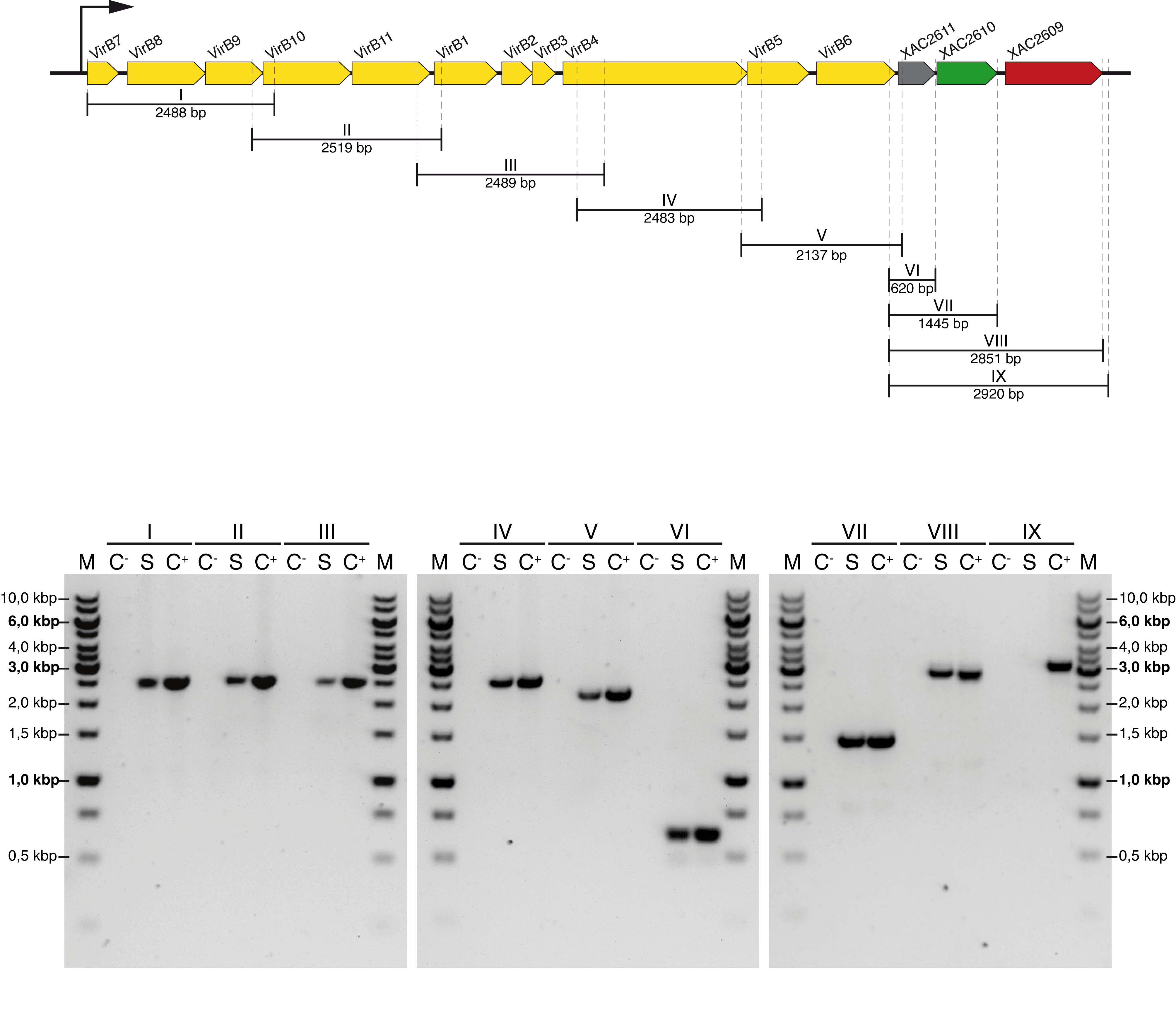

Supplement: S2 Fig — The upper panel shows a diagram of X. citri virB operon, from virB7 to xac2609. A black arrow indicates the promoter proposed for gene transcription, located upstream of virB7. Genes coloured in yellow correspond to structural T4SS components, in grey to hypothetical proteins, in green to X-Tfis, and in red to X-Tfes. Nine sets of primers (designated I to IX) amplifying overlapping regions were designed in order to determine the length of the polycistronic mRNA transcript (see Materials and Methods). The lower panel shows pictures of SYBR Safe stained 1% agarose gels in which the PCR products (depicted in the upper panel) were applied. C-: negative control, non-reverse transcribed X. citri RNA template; S: sample, reverse transcribed X. citri RNA template; C+: X. citri genomic DNA template. (TIF) [file ppat.1008561.s002.tif]

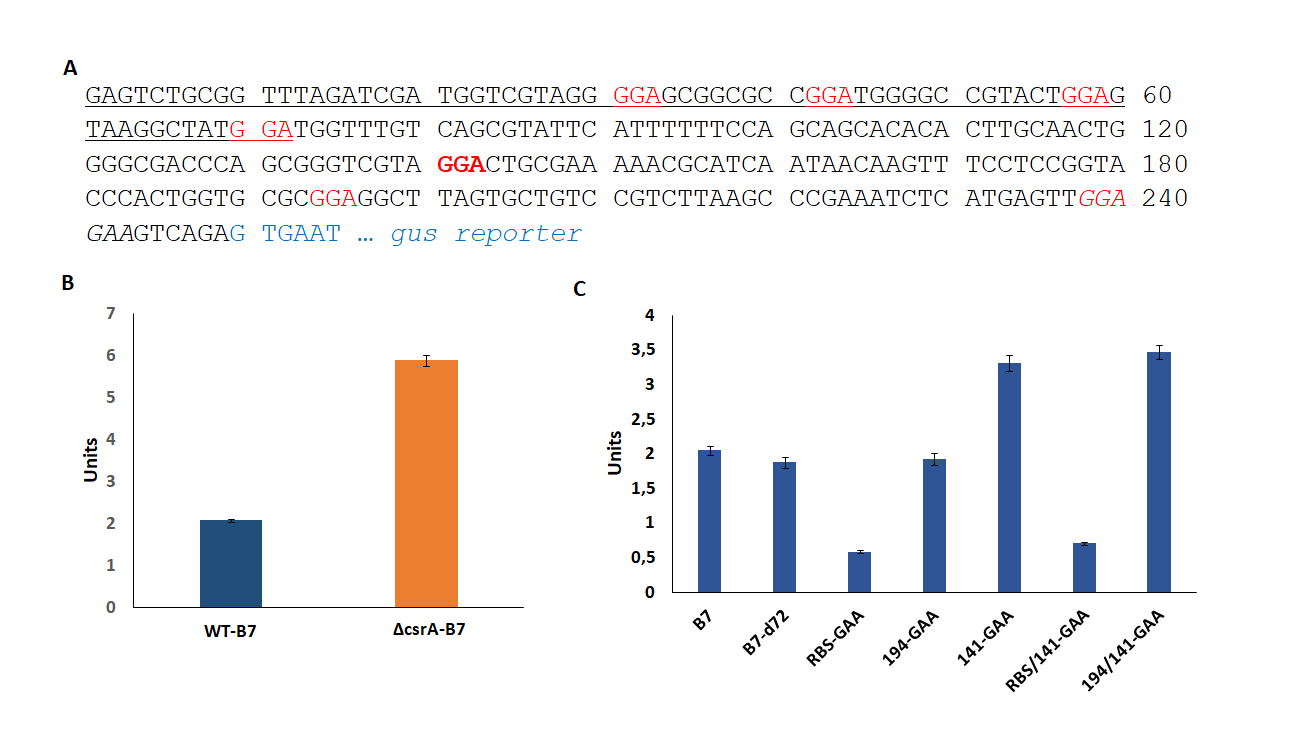

Supplement: S3 Fig — A) Nucleotide sequence of the 5'UTRB7 used in the amy::Ptac-5´UTRB7-gus reporter (translational fusion of the promoterless gusA in-frame fused to the 5´UTRB7 and first two codons of the virB7 gene under control of the constitutive Ptac promoter). Single underline: nucleotides 1–72. Italic type: putative ribosome binding site (RBS) at positions 238–243. Red type: GGA motifs at positions +31, +42, +57, +70, +141 (bold), +194 and +238 (within RBS). Blue type: beginning of coding region for gus reporter gene in which the first two virB7 codons were maintained. B) GUS activities of wild-type and csrA mutant cells harbouring the amy::Ptac-5´UTRB7-gus reporter. C) GUS activities of X. citri wild-type cells harbouring the amy::Ptac-5´UTRB7-gus reporter and its mutants. B7: wild-type 5´UTRB7. B7-d72: deletion of first 72 nucleotides of the 5´UTRB7. RBS-GAA: GGA to GAA mutation in the ribosome binding site (RBS) at position 238. 194-GAA: GGA to GAA at position 194. 141-GAA: GGA to GAA mutation at position 141. RBS/141-GAA: double mutant carrying both RBS-GAA and 141-GAA. 194/141-GAA, double mutant carrying both 141-GAA and 194-GAA. In B and C, Cells were cultured in nutrient both medium for 20 h and assayed for GUS activity. The quantification of GUS activity was performed using p-nitrophenyl β-D-glucuronide (PNPG) as substrate as described in Materials and Methods. Experiments were repeated three times. The means ± standard deviations are shown. (TIF) [file ppat.1008561.s003.tif]

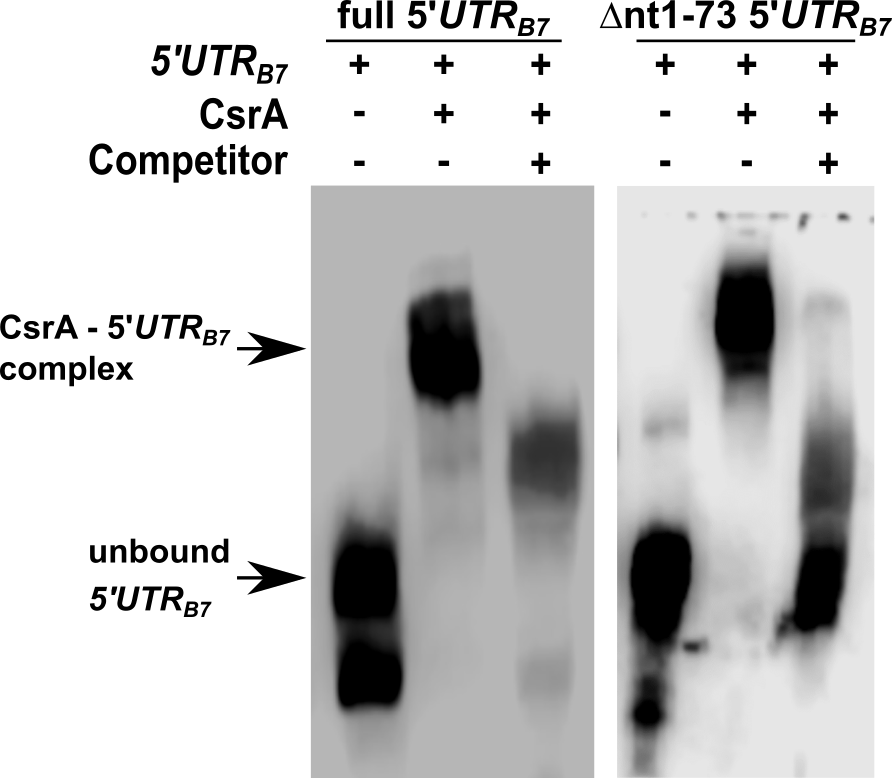

Supplement: S4 Fig — EMSA shows direct in vitro binding of CsrA with the complete 5´UTRB7 and a shortened Δnt1-73 5´UTRB7 fragment (lacking the first 73 nucleotides, up to and including the fourth GGA motif). See Fig 1 from the main text for the 5´UTRB7 sequence. Binding reactions were performed using 70 nM of purified CsrA protein and 6.25 nM Biotin-labelled RNA. Addition of 312.5 nM unlabelled 5´UTRB7 RNA competes with labelled RNA binding. See Materials and Methods for details. (TIFF) [file ppat.1008561.s004.tiff]

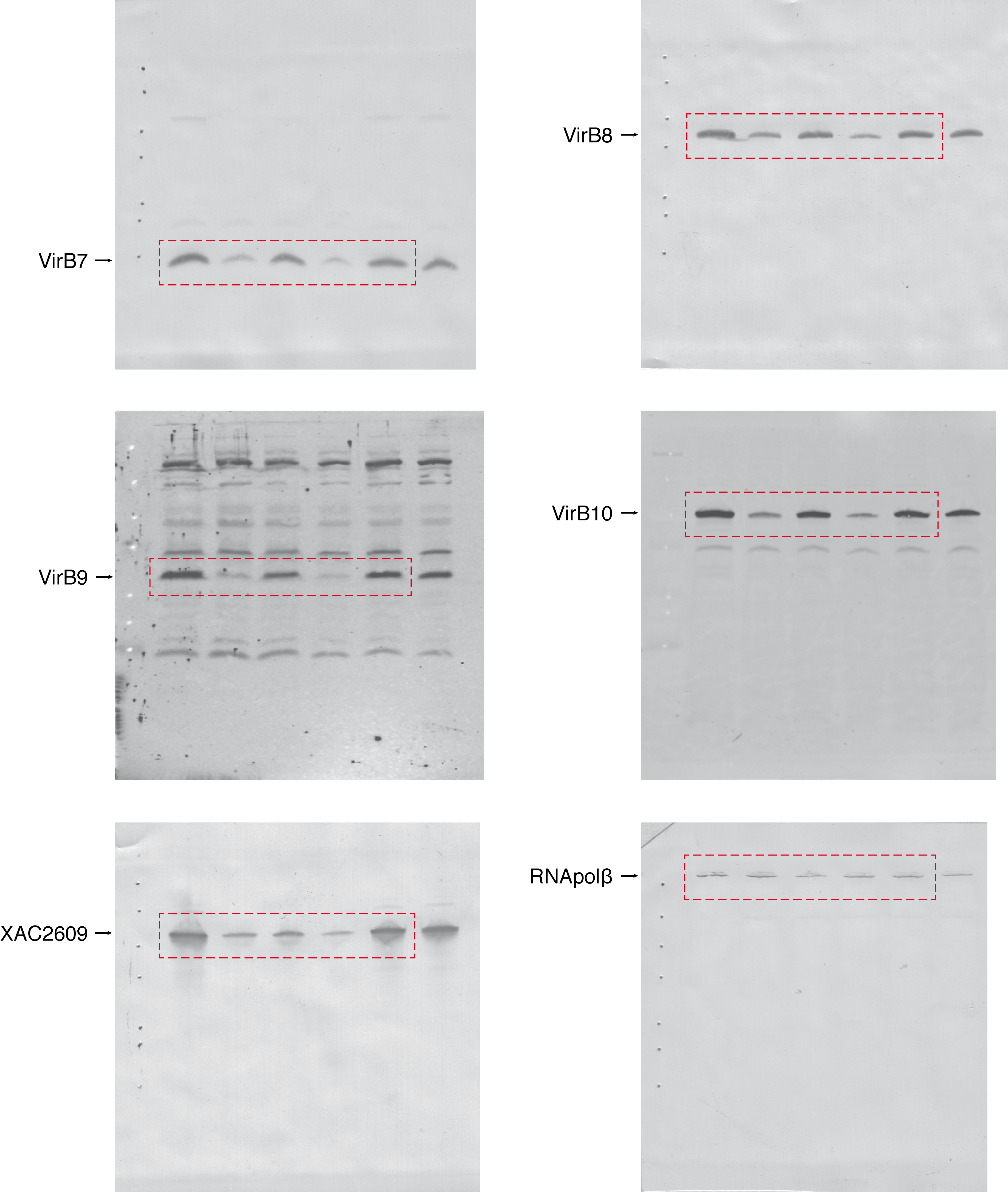

Supplement: S5 Fig — Polyclonal specific antibodies (Ab) against VirB7, VirB8, VirB9, VirB10, and XAC2609 were used. In the case of the β subunit of RNA polymerase (RNApolβ), monoclonal antibodies were used (see Materials and Methods for details). Experiments were repeated five times for VirB7, VirB8, VirB9, VirB10, and XAC2609 and four times for RNApolβ showing similar results. Expected molecular weights for mature proteins are: VirB7 12.62 kDa, VirB8 37.38 kDa, VirB9 26.56 kDa, VirB10 41.53 kDa, XAC2609 47.10 kDa, and RNApolβ 154.20 kDa. Note that VirB10 is a proline-rich protein, therefore it presents an apparent higher molecular weight. (TIFF) [file ppat.1008561.s005.tiff]

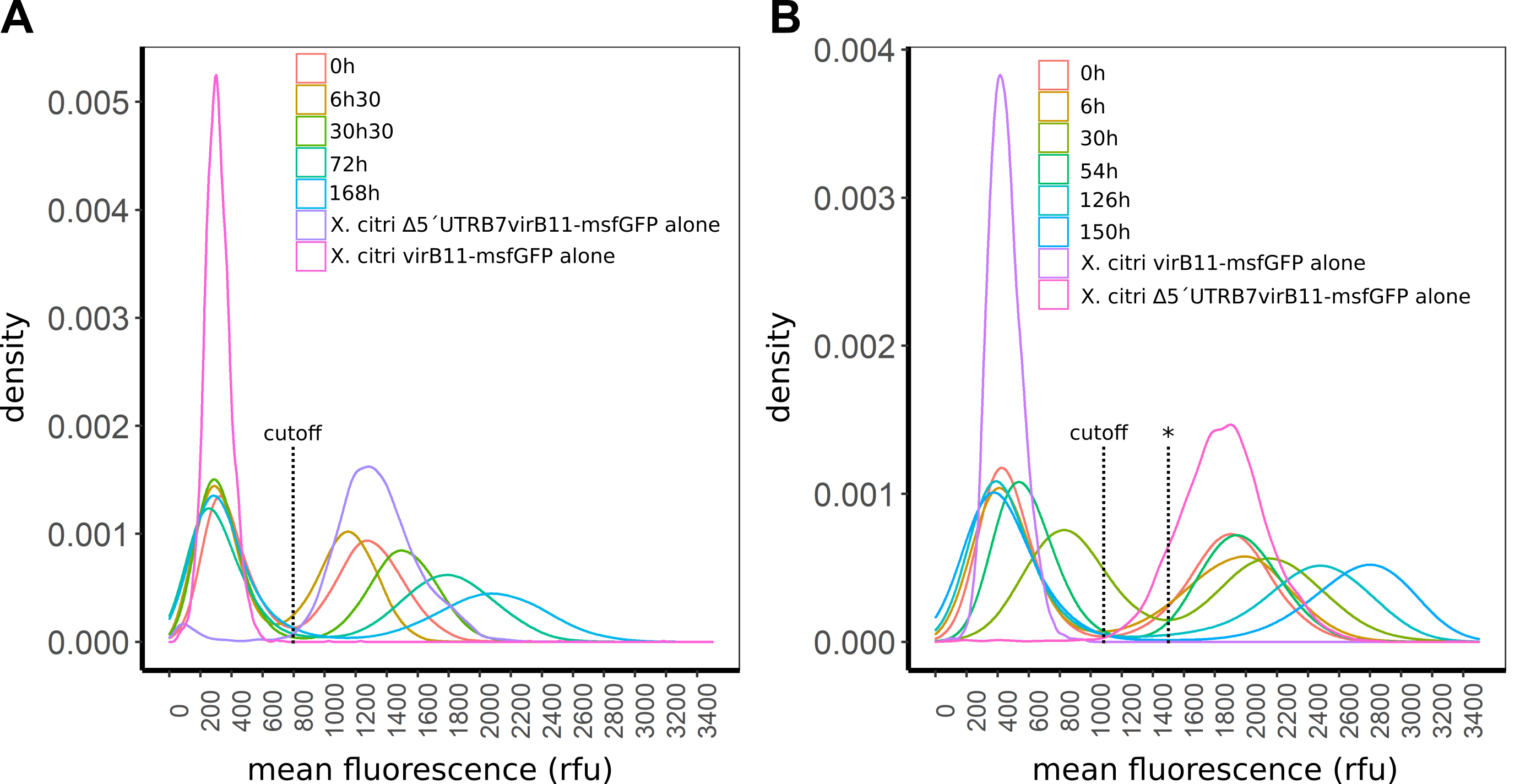

Supplement: S6 Fig — Shown are the distributions of the mean fluorescence levels for the two co-culture experiments of strains X. citri Δ5´UTRB7virB11-msfgfp and X. citri virB11-msfgfp at the different sampling timepoints. A) Mean fluorescence distributions for the first experiment represented by red lines in Fig 5 at timepoints 0h, 6h30, 30h30, 72h and 168h. Fluorescence intensity distribution of the two strains measured separately at time-point 0h are also shown (X. citri Δ5´UTRB7virB11-msfgfp alone and X. citri virB11-msfgfp alone). The cut-off value used for cell-sorting at all timepoints, 800 rfu, is shown. B) Mean fluorescence distributions from the represented by green lines in Fig 5 at timepoints 0h, 6h, 30h, 54h,126h and 150h. Fluorescence intensity distribution of the two strains measured separately at timepoint 0h are also shown (X. citri Δ5´UTRB7virB11-msfgfp alone and X. citri virB11-msfgfp alone). The cut-off value used for cell-sorting was 1100 rfu at all time points except for 30h where the cut-off used was 1500 rfu (indicated with an *). This discrepancy at 30h was very likely due to altered microscopy settings on that day of measurement but did not impair the efficiency sorting cells in this experiment. (TIFF) [file ppat.1008561.s006.tiff]
