# Supplementary material for: Bactericidal type IV secretion system homeostasis in Xanthomonas citri
Source: PLoS Pathog. 2020 May 26;16(5):e1008561. doi: 10.1371/journal.ppat.1008561 (PMC7286519; doi:10.1371/journal.ppat.1008561)
Supplement: S1 Table — (PDF) [file ppat.1008561.s007.pdf]

Table S1. Primers, strains and plasmids used in this study

Primer Cloning purpose	Primer name	Sequence 5' - 3'	Description
**Construct in pNPTS138 for transcriptional fusion *virB11-msfgfp***			
	CW003	TCAG **GAATTC** *GGAGTGAAACGATG* AGTAAAGGTGAAGAACTGTTCAC	Introduce ribosome binding site and start codon to *msgfp* in pDHL1029. Forward primer.
	CW004	TCAG **CCCGGG** TTATTTGTAGAGTTC	Introduce ribosome binding site and start codon to *msgfp* in pDHL1029. Reverse primer.
	CW005	TCAG **AAGCTT** CGCCTGACGTGACCG	Reverse primer 1. For upstream target. Ligates to pNPTS138.
	CW006	TCAG **GAATTC** CTAGCCGCCGCGCAG	Forward primer 1. For upstream target. Ligates to *msgfp* .
	CW007	TCAG **CCCGGG** GGCATCGAGTTCGTCGCTG	Reverse primer 2. For downstream target. Ligates to *msgfp* .
	CW008	TCAG **GGATCC** GTCCAGCAGCCATATCCTTG	Forward primer 2. For downstream target. Ligates to pNPTS138.
	CW048	TACAGT **GAATTC** GGAGTGAAAC	Forward primer for amplifying *msgfp* with ribosome binding site from pDHL-*RBS-msfgfp* .
	CW004	TCAG **CCCGGG** TTATTTGTAGAGTTC	Reverse primer for *amplifying msgfp* with ribosome binding site from pDHL-*RBS-msfgfp.*
	CW061	CCATGACGATCGATGACTC	Confirmation of genomic *virB11-msfgfp* construct. Forward primer.
	CW062	GCAATGCCTTTACTGCCTG	Confirmation of genomic *virB11-msfgfp* construct. Reverse primer.
**Construct in pNPTS138 for *csrA* deletion strain**			
	CW040	TATCAG **GAATTC** CCTGATCGACTGCGCTTTG	Forward primer 1. For downstream target. Ligates to pNPTS138.
	CW041	TTCATG **CTCGAG** TACCCGGCGAGTGAGGATC	Reverse primer 1. For downstream target. Ligates to upstream target.
	CW042	AATGTC **CTCGAG** TCGAATTGATCGTTGTCAAGG	Forward primer 2. For upstream target. Ligates to dosnwstream target.
	CW043	TTCATG **AAGCTT** GAACGCCGAACATCGATAGG	Reverse primer 2. For upstream target. Ligates to pNPTS138.
	CW072	GTACTGGGAACGCATGTGC	Confirmation of genomic deletion of *csrA* . Forward primer.
	CW073	GTCCTGAGTGCCATTCATGG	Confirmation of genomic deletion of *csrA* . Reverse primer.
**Construction of UTR_{B7} construct in between P_{TAC} and msfGFP in pPM7G**			
	CW244	**CTTCCTTTCCGTCAGCG** CCACACATTATACGAGCCGATG	Reverse outward primer for pPM7G. Includes the PTAC promotor. Homology for unstructered small RNA with ribosome binding site. Homology with primer CW246
	CW245	CTGGCGTTACCCAACTTAATCG	Forward outward primer fo pPM7G. Downstream of pPM7G cloned GFP construct.
	CW246	**CGCTGACGGAAAGGAAGGCTGGCGATG** AGTAAAGGTGAAGAACTGTTC	Amplifies msfGFP with unstructured small RNA. Homology with primer CW244. Includes start for GFP. Forward primer.
	CW247	**CGATTAAGTTGGGTAACGCCAG** *TTA* TTTGTAGAGTTCATCCATGC	Amplifies msfGFP with homology to primer CW245. Reverse primer
	CW248	CCACACATTATACGAGCCGATG	Reverse outward primer for pPM7G. Includes the PTAC promotor. Use with CW245.
	CW249	**ATTAATCATCGGCTCGTATAATGTGTGG** GAGTCTGCGGTTTAGATCGATG	Amplifies UTR_{B7} with homology to primer CW248. Forward primer.
	CW250	**ACCGGTGAACAGTTCTTCACCTTTACT** CACTCTGACTTCTCCAACTCATG	Amplifies UTR_{B7} with homology to primer CW30. Includes start site of *virB7* serving as start for GFP and *virB7* ribosome binding site. Reverse primer
	CW030	AGTAAAGGTGAAGAACTGTTC	Amplifies msfGFP with no start site. Forward Primer. Reverse primer is CW247.

	CW063	GCACCATCATCACCAATCAC	Control primer to confirm *virB* integrity. Forward primer.
	CW064	GCATTTGATCAGGAAATGCTG	Control primer to confirm *virB* integrity. Reverse primer
**Construct in pNPTS138 for deleting all *virB* genes and *xac2609* , *xac2610* and *xac2611,* replacing operon with msfGFP under control of P_{virB} with or without *UTR* $_{B7}$**			
	CW216	**AGGATATCTGGATCCACGAATTCGCTAG** CTAGGAGCAGTGTCGGTATCG	Amplifies downstream target. pNPTS138 homology. Reverse 1 primer. Use with CW217.
	CW217	**TCACGCACGGCATGGATGAACTCTACAAATAA** CGTTGCACTTCAGCGAGC	Amplifies downstream target. *msfGFP* homology. Forward 1 primer.
	CW239	ACCGGTGAACAGTTCTTCACCTTTACT **CAC**TCTGACTTCTCCAACTCATG	Amplifies upstream target. *msfGFP* homology. Reverse 2 primer. Places *msfGFP* start at *virB7* start.
	CW240	TCACTTAAGGCCTTGACTAGAGGGTCGA GACCTTCGCCTCGATCATG	Amplifies upstream target. pNPTS138 homology. Forward 2 primer.
	CW271	AGTTCTTCACCTTTACT CACTCTGACTTCTCCAA CTAAACCGCAGACTCTTGC	Amplifies upstream target. *msfGFP* homology. Reverse 2 primer. Also deletes UTR$_{B7}$ but maintains *virB7* ribosome binding site and *virB7* start codon for *msfGFP* . Use with primer CW240.
	CW031	TTTGTAGAGTTCATCCATGC	Amplifies *msfGFP* . Use with primer CW030. Assembles inbetween upstream and downstream targets.
	CW224	CAGAATCTGGCCCACACTG	Confirmation of genomic deletion of entire virB operon. Forward primer. Use CW063 as reverse primer.
**Construct in pNPTS138 for specifically deleting UTR$_{B7}$ leaving rest of *virB* operon intact**			
	CW277	**TCACTTAAGGCCTTGACTAGAGGGTCGA** GAGACCGTGTTCCAGTCAC	Forward primer 1. Amplifies downstream target. Homology to pNPTS138.
	CW278	**GGGCAAAGTTAGCAAGAGTCTGCGGTTTAG** GTTGGAGAAGTCAGAGTGAATC	Reverse primer 1. Amplifies downstream target. Homology to primer CW279.
	CW279	**ATACATCGGATTCACTCTGACTTCTCCAAC** CTAAACCGCAGACTCTTGC	Forward primer 2. Amplifies upstream target. Homology to primer CW278.
	CW315	**AGGATATCTGGATCCACGAATTCGCTAG** GACCTTCGCCTCGATCATG	Reverse primer 2. Amplifies upstream target. Homology to pNPTS138.
			Use primers CW063 and CW064 for control of genomic deletion
**Internal primers for 5´RACE assay**			
	SP1-D4	GCAACCCAAGCGCGCTGAAATAGC	Nested primers for cDNA production of the *virD* transcript.
	SP2-D4	GAGCCGCAGCAACAGCAGGGTCAG	
	SP3-D4	CCACCAGCGTGGCAATTGCGATGA	Third primer for final PCR of the *virD* cDNA, identifying the TSS by sequencing.
	SP1-B7	ACGACCACCAAAGTCAGGAGCAGGCT	Nested primers for cDNA production of the *virB* transcript.
	SP2-B7	GCACGCGCCTACCAATGCTGCAGC	
	SP3-B7	CAGCCACTAGCACCAACGACAGCTTG	Third primer for final PCR of the *virB* cDNA, identifying the TSS by sequencing.
**Primers for cloning UTR$_{B7}$ DNA fragments in pGEM-T**			
	F-B7	**TAATACGACTCACTATAGGG** AGTCTGCGGTTTAGATCGAT	Forward primer for PCR of the full length UTR$_{B7}$, containing the T7 promoter (bold font)
	F-B7-d73	**TAATACGACTCACTATAGGG** TGGTTTGTCAGCGTATTCATT	Forward primer for PCR of the shortened Δ1-73 UTR$_{B7}$, containing the T7 promoter (bold font)
	R-B7	ATCGGATTCACTCTGACTTCTCCAACTCATGA	Reverse primer for PCR of the different UTR$_{B7}$ fragments

**Overlapping analysis for determination of T4SS operon length**			
	RT_Pair1_F	TGAATCCGATGTATGTGTCCAAG	Amplify 2488 bp for overlapping PCR Pair I.
	RT_Pair1_R	GATCGGCTCATTGGCATCC	
	RT_Pair2_F	GTCTTCGCTCGCGAGAAAG	Amplify 2519 bp for overlapping PCR Pair II.
	RT_Pair2_R	TAGGGATTACGCGACGACTC	
	RT_Pair3_F	GGACCAGCTTGCGTTGATG	Amplify 2489 bp for overlapping PCR Pair III.
	RT_Pair3_R	AGAAAACCAGGCCGTTCTTTG	
	RT_Pair4_F	AACACGTTCAACCGTCTTCTG	Amplify 2483 bp for overlapping PCR Pair IV.
	RT_Pair4_R	TGGTCGTTCCATTGAGCTTG	
	RT_Pair5_F	CAATGGTTGGCCGATTTCTATG	Amplify 2137 bp for overlapping PCR Pair V.
	RT_Pair5_R	GGGCAGTCCAACTTACAAGAAG	
	RT_Pair6789_F	TCCTCAAAGGGCACGTGG	Amplify 620 bp, 1445 bp, 2851 bp and 2920 bp for overlapping PCR Pairs VI, VII, VIII and IX, respectively. All these pairs share the same forward primer.
	RT_Pair6_R	TCAATATTTCTTGAATATTGGATCAGAACAG	
	RT_Pair7_R	CTAGTTGGACACCTCCTTCATTTC	
	RT_Pair8_R	CCCGACTTACATGGACATTGAG	
	RT_Pair9_R	TAGAGCCAAGGCGGACTTC	
**RT-qPCR**			
	RT_16S_F	tgcaactcgactccatgaag	Amplify 113 bp of 16S (used as house-keeping gene).
	RT_16S_R	caacaaactcccatggtgtg	
	RT_VirD4_F	tcatcaacctgctcttcagc	Amplify 108 bp of *virD4.*
	RT_VirD4_R	aggtgaattcgtccatcagc	
	RT_VirB7_F	atgtgtccaagctgtcgttg	Amplify 107 bp of *virB7.*
	RT_VirB7_R	aaagtggttgacgtgcttcc	
	RT_VirB8_F	acaagaactccacggtttcg	Amplify 117 bp of *virB8.*
	RT_VirB8_R	gaaagcccagaggattttcc	
	RT_VirB9_F	ttacagcaccggtttcactg	Amplify 121 bp of *virB9.*
	RT_VirB9_R	ataagaatgcgtcgcggtac	
	RT_VirB10_F	atattcgctgcatcctggag	Amplify 124 bp of *virB10.*
	RT_VirB10_R	atcttggaccctttcggaag	
	RT_VirB11_F	tcgaggtgatcaaacgtctg	Amplify 92 bp of *virB11.*
	RT_VirB11_R	atcgatacccgtgatgaagc	
	RT_VirB1_F	taatccctacgccattggtg	Amplify 117 bp of *virB1.*
	RT_VirB1_R	cccagcgaaaagttgtaacc	
	RT_VirB2_F	gaccaagctgttgaacatgg	Amplify 116 bp of *virB2.*
	RT_VirB2_R	caatgagaattggcgagacc	
	RT_VirB3_F	tttcaggctgctgggattac	Amplify 108 bp of *virB3.*
	RT_VirB3_R	gggggattcttgcgatattc	
	RT_VirB4_F	acaacaatccatggggtgac	Amplify 110 bp of *virB4.*
	RT_VirB4_R	aatgagttctcggacggatg	
	RT_VirB5_F	aggaagagcgcaagaagatc	Amplify 106 bp of *virB5.*
	RT_VirB5_R	atctgctggcgatcgatatc	
	RT_VirB6_F	ggctagagaaacgcaggatac	Amplify 106 bp of *virB6.*

RT_VirB6_R	aagcccacgattacctgaac	
RT_2611_F	tgtcggaagataaggcgaag	Amplify 109 bp of *xac2611.*
RT_2611_R	gaaaccaaagctgcacactg	
RT_2610_F	atggccaacggtctattctc	Amplify 115 bp of *xac2610.*
RT_2610_R	tcagcactgcctcgttattc	
RT_2609_F	gatcgtcaaaaccgctcttg	Amplify 118 bp of *xac2609.*
RT_2609_R	gcgtccgaaatcttcttcag	

**Plasmid**	**Description/Purpose**	**Reference**
pNPTS138	Contains a kanamycin Resistance gene, SacB and a pMB1 ori (suicide in *X. citri*). Standard plasmid used for creating specific genomic insertions or deletions by 2-step alelic exchanged.	Howard Steinman (Addgene plasmid # 41891)
pPM7G	Contains a kanamycin Resistance gene, a pMB1 ori (suicide in *X. citri*) and homology to the *amy* gene in *X. citri* . Constructs cloned in the MCS together with the plasmid are integrated into the *amy* gene in *X. citri* .	Martins et al., 2010
pPM7G-P_{tac}-*msfgfp*	contains *msfgfp* under control of the P_{tac} promoter.	This study
pPM7G-P_{tac}-5'UTR$_{B7}$-*msfgfp*	contains *msfgfp* under control of the P_{tac} promoter with the 5'UTR$_{B7}$ inbetween P_{tac} and *msfgfp*	This study
pGEM-T	Contains the T7 promoter upstream of the MCS for *in vitro* production of RNA from cloned fragments	Promega

pDHL1029	Contains template for *msfgfp* with a flexible linker for translational fusions	Ke et al., 2016
pDHL-*RBS-msfgfp*	Contains template for *msfgfp* with a ribosome binding site and start site for transcriptional fusions	This study

Strain	Description/Purpose	Reference
Xanthomonas citri pv. citri str. 306	Wild-type *X. citri* strain	da Silva et al., 2002.
Escherichia coli str. K-12 substr. MC	*Lac* positive *E. coli* strain for competition assays	Hayashi et al., 2006.
Escherichia coli str. Dh5α	Transformation strain for cloning purposes	Taxonomy ID: 668369
X. citri virB10::msfgfp $_{TL}$	The T4SS´s ViB10 protein linked to msfGFP. Constructed in the native allele.	Sgro et al., 2018
X. citri virB11-msfgfp	fusion of *msfgfp* in between *virB11* and	This study
X. citri virB11-msfgfp ΔcsrA	*X. citri virB11-msfgfp* with *csrA* deletion	This study
X. citri amy::P $_{tac}$ *-msfgfp*	Insertion of pPM7G-*P* $_{tac}$ -	This study
X. citri amy::P $_{tac}$ *-5'UTR* $_{B7}$ *-msfgfp*	Insertion pPM7G-*P* $_{tac}$ *-5'UTR* $_{B7}$ -	This study
X. citri amy::P $_{tac}$ *-msfgfp ΔcsrA*	*X. citri amy::P* $_{tac}$ *-msfgfp* with *csrA* deletion	This study
X. citri amy::P $_{tac}$ *-5'UTR* $_{B7}$ *-msfgfp* [illegible]	*5'UTR* $_{B7}$ *-msfgfp*	This study
X. citri ΔvirB::P $_{B7}$ *-msfgfp*	Replacement of the entire *virB* operon (including effector pair *XAC2610* and *XAC2609*) with *msfgfp.* Placing the *msgfp* start cod	This study
X. citri ΔvirB::P $_{B7}$ *-Δ5'UTR* $_{B7}$ *-msfg*	*X. citri ΔvirB::P* $_{B7}$ *-msfgfp* with deletion of the 5'UTR$_{B7}$	This study
X. citri ΔvirB::P $_{B7}$ *-msfgfp ΔcsrA*	*ΔvirB::P* $_{B7}$ -	This study
X. citri ΔvirB::P $_{B7}$ *-Δ5'UTR* $_{B7}$ *-msfg*	*X. citri ΔvirB::P* $_{B7}$ *-Δ5'UTR* $_{B7}$ *-msfgfp* with *csrA* deletion	This study
X. citri Δ5'UTRB7 virB11-msfgfp	*X. citri virB11-msfgfp* with deletion of the 5'UTRB7	This study
