# Supplementary material for: Bactericidal type IV secretion system homeostasis in Xanthomonas citri
Source: PLoS Pathog. 2020 May 26;16(5):e1008561. doi: 10.1371/journal.ppat.1008561 (PMC7286519; doi:10.1371/journal.ppat.1008561)
Supplement: Attachment — Submitted filename: PPATHOGENS-D-20-00155_reviewer.pdf [file ppat.1008561.s010.pdf]

PLOS Pathogens

Bactericidal Type IV Secretion System Homeostasis in Xanthomonas citri

--Manuscript Draft--

**Manuscript Number:**	PPATHOGENS-D-20-00155
**Full Title:**	Bactericidal Type IV Secretion System Homeostasis in Xanthomonas citri
**Short Title:**	Bactericidal Type IV Secretion System Homeostasis
**Article Type:**	Research Article
**Section/Category:**	Plant Pathogens
**Keywords:**	Type IV secretion system, X-T4SS, bacterial competition, gene expression, homeostasis, Xanthomonas
**Abstract:**	Several Xanthomonas species have a type IV secretion system (T4SS) that injects a cocktail of antibacterial proteins into neighbouring Gram-negative bacteria, often leading to rapid lysis upon cell contact. This capability represents an obvious fitness benefit since it can eliminate competition while the liberated contents of the lysed bacteria could provide an increase in the local availability of nutrients. However, the production of this Mega Dalton-sized molecular machine, with over a hundred subunits, also imposes a significant metabolic cost. Here we show that the chromosomal virB operon, which encodes the structural genes of this T4SS in X. citri, is regulated by the conserved global regulator CsrA. Relieving CsrA repression from the virB operon produced a greater number of T4SSs in the cell envelope and an increased efficiency in contact-dependent lysis of target cells. However, this was also accompanied by a physiological cost leading to reduced fitness when in co-culture with wild-type X. citri. We show that T4SS production is constitutive despite being downregulated by CsrA. Cells subjected to a wide range of rich and poor growth conditions maintain a constant density of T4SSs in the cell envelope and concomitant interbacterial competitiveness. These results show that CsrA provides a constant though partial repression on the virB operon, independent of the tested growth conditions, in this way controlling T4SS-related costs while at the same time maintaining X. citri's aggressive posture when confronted by competitors.
**Additional Information:**	
**Question**	**Response**
**Financial Disclosure**<br><br>Enter a financial disclosure statement that describes the sources of funding for the work included in this submission. Review the submission guidelines for detailed requirements. View published research articles from *PLOS Pathogens* for specific examples.<br><br>This statement is required for submission and **will appear in the published article** if the submission is accepted. Please make sure it is accurate.	This work was supported by FAPESP - Fundação de Amparo a Pesquisa do Estado de São Paulo (http://www.fapesp.br/) through post-doctoral scholarships to WC (2015/18237-2), GGS (2014/04294-1), and EL (2019/12234-2) and research grants to CSF (2011/07777-5 and 2017/17303-7).<br>The funders had no role in study design, data collection and analysis, decision to publish, or preparation of the manuscript.

Powered by Editorial Manager® and ProduXion Manager® from Aries Systems Corporation

**Unfunded studies**<br>Enter: *The author(s) received no specific funding for this work.*<br><br>**Funded studies**<br><br>Enter a statement with the following details:<br>• Initials of the authors who received each award<br>• Grant numbers awarded to each author<br>• The full name of each funder<br>• URL of each funder website<br>• Did the sponsors or funders play any role in the study design, data collection and analysis, decision to publish, or preparation of the manuscript?<br>• **NO** - Include this sentence at the end of your statement: *The funders had no role in study design, data collection and analysis, decision to publish, or preparation of the manuscript.*<br>• **YES** - Specify the role(s) played.<br><br>* typeset	
**Competing Interests**<br><br>Use the instructions below to enter a competing interest statement for this submission. On behalf of all authors, disclose any competing interests that could be perceived to bias this work—acknowledging all financial support and any other relevant financial or non-financial competing interests.<br><br>This statement **will appear in the published article** if the submission is accepted. Please make sure it is accurate. View published research articles from *PLOS Pathogens* for specific examples.	The authors have declared that no competing interests exist.

**NO authors have competing interests**<br><br>Enter: *The authors have declared that no competing interests exist.*<br><br>**Authors with competing interests**<br><br>Enter competing interest details beginning with this statement:<br><br>*I have read the journal's policy and the authors of this manuscript have the following competing interests: [insert competing interests here]*<br><br>* typeset	
**Data Availability**<br><br>Authors are required to make all data underlying the findings described fully available, without restriction, and from the time of publication. PLOS allows rare exceptions to address legal and ethical concerns. See the PLOS Data Policy and FAQ for detailed information.<br><br>A Data Availability Statement describing where the data can be found is required at submission. Your answers to this question constitute the Data Availability Statement and **will be published in the article**, if accepted.<br><br>**Important:** Stating 'data available on request from the author' is not sufficient. If your data are only available upon request, select 'No' for the first question and explain your exceptional situation in the text box.<br><br>Do the authors confirm that all data underlying the findings described in their manuscript are fully available without restriction?	Yes - all data are fully available without restriction
**Describe where the data may be found in full sentences. If you are copying our sample text, replace any instances of XXX with the appropriate details.**<br>• If the data are **held or will be held in a**	All relevant data are within the manuscript and its Supporting Information files.

**public repository**, include URLs, accession numbers or DOIs. If this information will only be available after acceptance, indicate this by ticking the box below. For example: *All XXX files are available from the XXX database (accession number(s) XXX, XXX.*).<br>• If the data are all contained **within the manuscript and/or Supporting Information files**, enter the following: *All relevant data are within the manuscript and its Supporting Information files.*<br>• If neither of these applies but you are able to provide **details of access elsewhere**, with or without limitations, please do so. For example:<br><br>*Data cannot be shared publicly because of [XXX]. Data are available from the XXX Institutional Data Access / Ethics Committee (contact via XXX) for researchers who meet the criteria for access to confidential data.*<br><br>*The data underlying the results presented in the study are available from (include the name of the third party and contact information or URL).*<br>• This text is appropriate if the data are owned by a third party and authors do not have permission to share the data.<br><br>* typeset	
Additional data availability information:	

Powered by Editorial Manager® and ProduXion Manager® from Aries Systems Corporation

Manuscript plus Figures and Supporting Figures and Table
Click here to access/download;Manuscript;Cenens_et_al_PLoS_Pathogens

Bactericidal Type IV Secretion System Homeostasis in Xanthomonas citri

William Cenens[1], Maxuel O. Andrade[2], Edgar Llontop[1], Cristina E. Alvarez-Martinez[3], Germán G. Sgro[1] and Chuck S. Farah[1]

Affiliations: [1]Departamento de Bioquímica, Instituto de Química, Universidade de São Paulo (USP), São Paulo, SP, Brazil. [2]Laboratório Nacional de Biociências, Centro Nacional de Pesquisa em Energia e Materiais, R. Giuseppe Máximo Scolfaro 10000, Campinas, SP, 13083-970, Brazil. [3]Departamento de Genética, Evolução, Microbiologia e Imunologia, Instituto de Biologia, Universidade Estadual de Campinas (UNICAMP), Rua Monteiro Lobato, 255, Campinas, SP, 13083-862, Brazil.

Contact: w.cenens@gmail.com and chsfarah@iq.usp.br

Abstract

Several *Xanthomonas* species have a type IV secretion system (T4SS) that injects a cocktail of antibacterial proteins into neighbouring Gram-negative bacteria, often leading to rapid lysis upon cell contact. This capability represents an obvious fitness benefit since it can eliminate competition while the liberated contents of the lysed bacteria could provide an increase in the local availability of nutrients. However, the production of this Mega Dalton-sized molecular machine, with over a hundred subunits, also imposes a significant metabolic cost. Here we show that the chromosomal *virB* operon, which encodes the structural genes of this T4SS in *X. citri*, is regulated by the conserved global regulator CsrA. Relieving CsrA repression from the *virB* operon produced a greater number of T4SSs in the cell envelope and an increased efficiency in contact-dependent lysis of target cells. However, this was also accompanied by a physiological cost leading to reduced fitness when in co-culture with wild-type *X. citri*. We show that T4SS production is constitutive despite being downregulated by CsrA. Cells subjected to a wide range of rich and poor growth conditions maintain a constant density of T4SSs in the cell envelope and concomitant interbacterial competitiveness. These results show that CsrA provides a constant though partial repression on the *virB* operon, independent of the tested growth conditions, in this way controlling T4SS-related costs while at the same time maintaining *X. citri*'s aggressive posture when confronted by competitors.

Key Words: Interbacterial competition, Type IV secretions systems, CsrA, Single-cell fluorescent microscopy

Author Summary

Xanthomonas citri is a member of a family of phytopathogenic bacteria that can cause substantial losses in crops. At different stages of the infection cycle, these cells will encounter other bacterial species with whom they will have to compete for space and nutrients. One mechanism which improves a cell´s chance to survive these encounters is a type IV secretion system that transfers a cocktail of antimicrobial effector proteins into other Gram-negative bacteria in a contact-dependent manner. Here, we show that this system is constitutively produced at a basal level, even during low nutrient conditions, despite representing a significant metabolic burden to the cell. The conserved global regulator, CsrA, provides a constant, nutrient-independent, repression on the production of T4SS components, thereby holding production costs to a minimum while at the same time ensuring *X. citri´*s competitiveness during encounters with bacterial rivals.

Introduction

Type IV secretion systems (T4SS) are large multiprotein nanomachines spanning both the inner and outer membrane of many Gram-negative bacterial species (Low et al., 2014). The best described functions of T4SSs are the delivery of the T-DNA of *Agrobacterium tumefaciens* (Gordon and Christie, 2014; Vergunst et al., 2000), their roles in bacterial conjugation (Ilangovan et al., 2015) and their involvement in delivering virulence factors to mammalian cells by several pathogenic species (Dehio and Tsolis, 2017). T4SSs found in Gram-negative bacteria are made up of over 100 subunits of 12 different proteins (VirB1 to VirB11 plus VirD4) with a total size of over 3 MDa each (Low et al., 2014) though some bacterial species carry systems with extra components and are much larger in size (Chung et al., 2019; Ghosal et al., 2019). Maintaining and expressing large operons and producing the

amino acids required to assemble the proteins they encode present a significant investment in terms of energy and raw materials for a cell (Akashi and Gojobori, 2002; Lynch and Marinov, 2015; Wagner, 2005). Given the high cost secretion systems would have on cell physiology, it is not surprising that their production is often restricted to specific conditions, where they will be most needed. For example, expression of the *A. tumefaciens* T4SS is dependent on pH, monosaccharides, phosphate and specific phenolic compounds released by wounded plant tissue (Das and Pazour, 1989; Gao and Lynn, 2005; Lohrke et al., 2001; Winans, 1990). Similarly, plasmid-borne *tra* genes, encoding a T4SS and other components of the conjugation machinery, are only produced during specific conditions and often only in a small portion of the population (Koraimann and Wagner, 2014). Other examples include the *Brucella suis* T4SS produced inside acidic phagocytic vacuoles of macrophages (Boschiroli et al., 2002) and the *Ehrlichia ruminantium* T4SS whose genes are induced during iron starvation (Moumène et al., 2017). This strict environmentally-dependent regulation is also common in other secretion systems; for example, the *Vibrio cholera* type VI secretion system (T6SS) is induced during high cell densities on chitinous surfaces (Borgeaud et al., 2015), the *Xanthomonas citri* T6SS is specifically induced in the presence of amoeba (Bayer-Santos et al., 2018), the *Shigella flexneri* type III secretion system (T3SS) is tightly regulated by oxygen levels (Marteyn et al., 2010) and T3SS expression in *Xanthomonas* species is induced upon interaction with their plant hosts (Schulte and Bonas, 1992; Zimaro et al., 2014).

X. citri is a phytopathogen that causes citrus canker, a disease which can lead to significant losses in citrus fruit production (Mansfield et al., 2012). Previously, our group has characterized the interbacterial killing activity of a T4SS in *X. citri* and showed that this strain actively transfers a cocktail of antibacterial effector proteins into neighbouring Gram-negative cells in a contact-dependent manner (Sgro et al., 2019; Souza et al., 2015). More recently, we also described the antibacterial killing of a similar T4SS with its unique antibacterial effectors in the opportunistic pathogen *Stenotrophomonas maltophilia* (Bayer-Santos et al., 2019) Despite the importance of T4SSs in interbacterial competition, little is known concerning the regulation of these Xanthomonadales-like T4SSs (X-T4SSs).

Microarray data of an *X. citri* strain harbouring a mutation in the global regulator CsrA (also called RsmA) indicated its involvement in the regulation of over a hundred genes, including the *virB* operon that encodes the T4SS proteins VirB1-11 (Andrade et al., 2014).

CsrA is a pleiotropic regulator linked to the genetic changes during stationary phase growth, biofilm formation, gluconeogenesis and virulence (Romeo and Babitzke, 2018). CsrA acts by binding specific mRNA loops in 5´ untranslated regions containing the canonical 5´-GGA-3´ motif (Holmqvist et al., 2016; Liu and Romeo, 1997). In some cases, these interactions stabilize the mRNA leading to increased expression, as for example has been observed for the *hrpG* mRNA in *X. citri* (Andrade et al., 2014). More often, these CsrA-binding loops encompass the ribosome binding site, in which case binding of CsrA inhibits translation (Baker et al., 2002). Although several other means of CsrA regulation exist (Romeo and Babitzke, 2018), the majority of interactions lead to a repression of protein production (Potts et al., 2017). CsrA/RsmA is regulated by two important small RNAs, CsrB and CsrC (in *Escherichia coli*) or RsmY and RsmZ (in *Pseudomonas aeruginosa*), which contain several high affinity CsrA binding loops that effectively titrate CsrA (Janssen et al., 2018; Weilbacher et al., 2003). Production of these small RNAs in *E. coli* is controlled by several regulatory pathways, including the BarA/UvrY two-component system that responds to molecules such as formate and acetate, the catabolite repression pathway mediated by cAMP-CRP and the stringent response governed by RelA and SpoT-mediated production of (p)ppGpp (Romeo and Babitzke, 2018). Furthermore, direct regulation of CsrA copy numbers in *E. coli* is achieved by five different promoters using at least two different sigma factors (Yakhnin et al., 2011).

X. citri CsrA is very similar to CsrA from *E. coli* and *P. aeruginosa* (>77% identical), albeit the *X. citri* protein has a 9 residue C-terminal extension. Detailed knowledge of CsrA, its targets and its regulation in *Xanthomonas* species, whose genomes do not code for CsrB and CsrC homologs, is limited (Abendroth et al., 2014). Nonetheless, some studies have shown phenotypic alterations in a CsrA deletion strain reminiscent with known CsrA phenotypes in *E. coli* and *P. aeruginosa*, such as reduced virulence, increased biofilm formation and increased glycogen production (Chao et al., 2008; Lu et al., 2012) and direct RNA binding studies have also confirmed the affinity of *X. citri* CsrA for the canonical 5´-GGA-3´ motifs (Andrade et al., 2014).

In this work, we show that CsrA from *X. citri* represses the *virB* operon and that the removal of this repression has a measurable fitness cost. However, CsrA repression is incomplete, and so production of the *virB* products continues at a controlled basal level, maintaining a constant density of T4SSs in the cell envelope during different growth

conditions. This sustained and energetically affordable aggressive posture contributes to *X. citri* competiveness and survival.

Results

CsrA regulates the virB operon by binding to the 5´UTR of virB7

Based on transcription start site analysis data for *Xanthomonas campestris* (Alkhateeb et al., 2016) and further observations made in the Sequence Read Archive for *X. campestris and X. citri* (https://www.ncbi.nlm.nih.gov/sra), the Xanthomonas *vir* locus contains two main transcription start sites (TSSs) (Figure 1A). The presence of these TSSs in *X. citri* was confirmed by 5´RACE analysis (Supplementary Figure S1). The first TSS is located 303 nucleotides upstream of the *virD4* start codon and a second TSS is located 249 nucleotides upstream of the *virB7* start codon (Figure 1A and Supplementary Figure S1). Thus, both *virD4* and *virB7* contain large upstream regions, with the upstream region of *virD4* containing an open reading frame that encodes a conserved protein of unknown function. Although an open reading frame can also be detected between *virD4* and *virB7* (nucleotide sequence shown in Figure 1A), it lacks a canonical ribosome binding site and its translated product is of very low sequence complexity and is not conserved in the protein databases. The organization of the open reading frames in the *virB* locus (*virB7-virB11* followed by *virB1-virB6*) plus three more downstream open reading frames (*xac2611*, *xac2610* and *xac2609*) suggest that they may be expressed as a polycistronic mRNA (Figure 1A and Supplementary Figure 2A). To test this hypothesis, *X. citri* RNA was purified and reverse-transcribed to produce cDNA which was then used as a template in overlapping PCR assays. Employing a set of nine specific primer pairs, we were able to show that a single polycistronic mRNA (approximately 13.7 kbp in length) is produced that encompasses all the genes from *virB7* to *xac2609* but does not extend beyond *xac2609* (Supplementary Figure 2B). We note that *xac2609* and *xac2610* code for a Xanthomonadaceae type IV secretion system effector (X-Tfe^{XAC2609}) previously shown to be secreted by the X-T4SS and its cognate immunity protein (X-Tfi^{XAC2610}) (Souza et al. 2015).

The list of CsrA-regulated genes in *X. citri* from a microarray dataset includes several of the *virB* genes (Andrade et al., 2014). We therefore further scrutinised the *virB* 5´UTR

(upstream of *virB7* start codon, from here on referred to as *5´UTR*$_{B7}$). This analysis revealed several 5´-GGA-3´ motifs (Figure 1A) that could be CsrA binding sites when present in a stable loop structure (Holmqvist et al., 2016). To test this hypothesis, a transcriptional *msfgfp* (encoding for the monomeric super folder Green Fluorescent Protein) fusion was constructed downstream of the genomic copy of *virB11* (*X. citri virB11-msfgfp*), located at the centre of the *virB* operon. Single-cell fluorescence analysis showed that upon deleting *csrA* in *X. citri virB11-msfgfp,* msfGFP production from the *virB* operon is upregulated 3.3-fold (Figure 1B). In order to confirm the direct regulation of CsrA on the *5´UTR*$_{B7}$, this region was cloned in between the P$_{tac}$ promoter (constitutive in the *lac* negative *X. citri* strain) and *msfgfp*, after which a single copy of this construct was integrated into the α-amylase gene (*amy*; *xac0798*) in both the *X. citri* wild-type and Δ*csrA* strains. Measurements of the fluorescence levels in thousands of individual cells that CsrA is capable of repressing msfGFP production from the P$_{tac}$ promoter only when the *5´UTR*$_{B7}$ is present, but not in its absence (Figure 1C). This indicates that the effect of CsrA is independent of the *virB* promoter. Expanding these observations, we replaced the entire structural *virB* operon (Figure 1A) with *msfgfp,* to produce the *X. citri* Δ*virB::P*$_{B7}$*-msfgfp* strain (with the *msfgfp* start codon in the exact position of the *virB7* start codon). Deletion of *csrA* in this strain produced a similar increase in msfGFP production (Figure 1D). Importantly, the removal of the *5´UTR*$_{B7}$ in this strain (*X. citri* Δ*virB::P*$_{B7}$*-Δ5´UTR*$_{B7}$*-msfgfp*) caused msfGFP levels to increase in the wild-type strain (Figure 1d). This increase in msfGFP production in the absence of *5´UTR*$_{B7}$ was only slightly lower than that observed in the *X. citri* Δ*virB::P*$_{B7}$*-msfgfp* Δ*csrA* and *X. citri* Δ*virB::P*$_{B7}$*-Δ5´UTR*$_{B7}$*-msfgfp* Δ*csrA* strains (Fig. 1d), further confirming CsrA regulation mediated by the *5´UTR*$_{B7}$. We note that the Δ*csrA* background has an elaborate effect on *X. citri* physiology (for instance, cultures display cellular flocculation and decreased cell-sizes) that could account for the subtle differences in msfGFP production observed between strains lacking *csrA* with or without the *5´UTR*$_{B7}$ in Figure 1d. Next, the construction of a genomic deletion of the *5´UTR*$_{B7}$, while keeping all other *virB* genes intact, resulted in a 3.9-fold increase in expression levels in the *X. citri* Δ*5´UTR*$_{B7}$ *virB11-msfgfp* reporter strain (Figure 1E). Finally, an electrophoretic mobility shift assay (EMSA) confirmed the direct *in vitro* binding of CsrA to the *5´-UTR*$_{B7}$ (Supplementary Figure 3). Taken together, the results presented so far suggest that CsrA regulates *virB* operon expression by binding to the *5´UTR*$_{B7}$, most probably by preventing translation and/or destabilizing the *virB* transcripts.

Since the results shown in Figure 1 are based on the fluorescence intensity of cells expressing an *msfGFP* reporter gene, we sought to observe the direct effect of CsrA and the *5´UTR*$_{B7}$ on *vir* mRNA and protein levels. Figure 2A shows quantitative real-time PCR experiments in which the relative levels of transcripts for all the open reading frames from *virD4* to *xac2609* in the *ΔcsrA* and *Δ5´UTR*$_{B7}$ *virB11-msfgfp* strains are presented with respect to the levels observed in the wild-type strain. Deletion of *csrA* resulted 8 to 16-fold increases in the amount of message observed for *virD4*, all of the *virB* open reading frames as well as the three downstream reading frames (*xac2611*, *xac2610* and *xac2609*), consistent with microarray data previously reported for the Δ*csrA* strain (Andrade et al., 2014). Deletion of the *5´UTR*$_{B7}$ also resulted in significant increases in the amount of message containing the first ten open reading frame of the *virB* operon (*virB7-virB11* plus *virB1-virB5*) but not for the last four (*virB6*, *xac2611*, *xac2610*, *xac2609*) (Figure 2A). As expected, the upstream *virD4* transcript levels did not increase in the *5´UTR*$_{B7}$ *virB11-msfgfp* background (Figure 2A). We performed western blots to analyse the levels (in total cell lysates) of the VirB7, VirB8, VirB9 and VirB10 components of the X-T4SS, coded by the first four open reading frames of the *virB* operon, and of X-Tfe^{XAC2609}, coded by the last open reading frame. Figures 2B and 2C and Supplementary Figure 4 show that the *virB11-msfgfp* reporter strain and the *X. citri* wild-type strain (1.0 fold-change baseline) have very similar levels of all five proteins while the deletion of *csrA* in either wild-type or *virB11-msfgfp* backgrounds results in 2.2 to 7.3-fold increases in all of these proteins. Deletion of the *5´UTR*$_{B7}$ in the *virB11-msfgfp* reporter strain also resulted in 2.2 to 5.4-fold increases in the detected levels of VirB7, VirB8, VirB9 and VirB10 while X-Tfe^{XAC2609} levels increased by a more modest factor of 1.8-fold (Figure 2B and 2C). Thus, a variety of experimental approaches (Figures 1 and 2) point to a direct role of CsrA and the *5´UTR*$_{B7}$ in the repression of the *virB* operon.

*Removal of CsrA/*5´UTR$_{B7}$ *repression on the virB operon increases T4SS numbers and bacterial killing*

We then asked whether the absence of the *5´UTR*$_{B7}$ would result in an increased number of assembled X-T4SSs in the bacterial envelope and a consequent enhancement in interbacterial killing efficiency. For these assays we took advantage of an *X. citri virB10-msfgfp*$_{TL}$ translational fusion strain (Sgro et al., 2018) in which the periplasmic VirB10

component has been replaced by a VirB10-msfGFP chimera. Since each T4SS contains 14 copies of VirB10, assembled T4SSs can be observed as fluorescent periplasmic foci and counted (see Materials and Methods and Sgro et al. 2018). Deleting the *5´UTR*$_{B7}$ in the *X. citri virB10-msfgfp*$_{TL}$ genetic background resulted in a 2.6-fold increase in the number of fluorescent T4SS foci that were counted per cell (Figure 3A). We note that the higher density of T4SSs in this strain leads to a more crowded periplasm, making it more difficult to clearly separate individual foci, leading to an underestimation of total number of X-T4SSs. Therefore, the calculated 2.6-fold increase should be considered a lower limit.

As could be expected, a greater number of T4SSs in the *X. citri Δ5´UTR*$_{B7}$*virB10-msfgfp*$_{TL}$ strain also increased the efficiency with which *X. citri* lyses *E. coli* cells in a quantitative LacZ mediated CPRG-cleavage assay (Figure 3B). Using the slopes of the curves in the CPRG-cleavage assays as a measure of killing efficiency (see Materials and Methods and Sgro et al. 2018), this strain kills 2.1-fold more efficiently than the *X. citri virB10-msfgfp*$_{TL}$ strain (Figure 3B). However, these results also show that under these conditions, removal of CsrA repression was not necessary to observe X-T4SS dependent *E. coli* lysis in the CPRG assays (Figure 3B), in agreement with previously published spot assays and CFU-based competition assays, all performed with wild-type *X. citri* strains (Souza et al., 2015). To test whether we could identify conditions in which X-T4SS-mediated killing would be inhibited or enhanced, we tested *E. coli* lysis efficiencies at 18°C or 28°C, at pH 6.0, 7.0 and 8.0, in the absence of Fe^{3+} and using different carbohydrate sources (glucose, sucrose or starch; Figure 3C). All the tested conditions led to clearly detectable T4SS-dependent lysis of *E. coli* cells with only small to moderate variations (+/- 35%) in killing efficiencies (Figure 3C). Taken together, these observations indicate that the observed levels of msfGFP signal from the *X. citri virB11-msfgfp* reporter strain (Figure 1A, 1B and 1E) and the discrete numbers of T4SSs in the cell envelope (Figure 3A) present in the wild-type background, represent the baseline expression and production levels of T4SS components and that these levels, all repressed from what they would otherwise be in the absence of CsrA, are sufficient to maintain X-T4SS production and efficient killing of neighbouring target cells.

X-T4SS dependent interbacterial killing is maintained during growth under scarce nutrient conditions

Considering the expected high energetic cost of producing T4SSs and the previously described responsiveness of *csrA* regulons to nutrient input (Romeo and Babitzke, 2018), we decided to test whether lowering the nutrient contents of the growth media would change T4SS-dependent killing efficiencies. To accomplish this, *X. citri* cultures were transferred to media containing normal or reduced levels of sucrose and/or casamino acids (the only nutrient sources present in the defined media) and grown overnight followed by an additional nine hours in fresh media for each culture to fully adapt to the conditions (see Materials and Methods for details). These cultures were then subjected to a quantitative CPRG-cleavage assay under standardized conditions (AB medium + 0.2% sucrose + 0.2% casamino acids). Figure 3D shows that *X. citri* grown in nutrient-scarce culture media continues to sustain T4SS-dependent lysis of *E. coli* cells. *X. citri* cultures with limited access to sucrose have slightly reduced killing efficiencies (23% decrease) while cells with limited access to both casamino acids and sucrose presented slightly elevated (39% increase) killing efficiencies (Figure 3D). Supplementary Movie 1 presents a time-lapse video of a mixed culture of *X. citri* and *E. coli* cells growing in casamino acid-depleted media (AB medium+ 0.2% sucrose + 0.01% casamino acids) for over 30 hours in which many events of *E. coli* cell lysis are observed upon contact with *X. citri* cells. For comparison, Supplementary Movie 2 presents *X. citri* killing *E. coli* cells under standard nutrient concentrations (AB + 0.2% sucrose + 0.2% casamino acids), and also shows clear cell lysis upon contact with *X. citri* cells. No *E. coli* lysis is observed in these experiments when X-T4SS-deficient *X. citri* or *S. maltophilia* strains are employed (Bayer-Santos et al., 2019; Sgro et al., 2018; Souza et al., 2015).

Constant but incomplete CsrA-mediated repression of the virB operon during different growth conditions

Figures 1E and 3A, showed that removing the $5'UTR_{B7}$ leads to both increased msfGFP production and T4SS assembly. In order to test how CsrA regulation impacts protein production from the *virB* operon, we decided to look more closely at VirB production in the presence and absence of the $5'UTR_{B7}$ under different growth conditions. To do this, we began by comparing cytoplasmic msfGFP production from *X. citri virB11-msfgfp* and *X. citri*

Δ5´UTR$_{B7}$ *virB11-msfgfp* strains. Both strains were grown simultaneously in media with sucrose, glucose, glycerol or starch as carbohydrate sources or in media depleted for casamino acids and/or sucrose. Cultures were grown overnight in the different media, diluted and grown for additional 6 hours in fresh media before msfGFP content was registered by fluorescence microscopy.

Mean msfGFP production from the *X. citri virB11-msfgfp* strain remains fairly similar during different inputs of carbohydrates and even in the absence of a carbohydrate source (Figure 4A; white boxplots). Reducing casamino acid concentrations, however, increases VirB production by 60% on average (both in combination with 0.2% or 0.01% sucrose). This increase in expression levels seems to be independent of CsrA regulation since similar increases in msfGFP production were detected when using the *X. citri Δ5´UTR*$_{B7}$ *virB11-msfgfp* strain (Figure 4A, dark grey boxplots). In fact, when comparing the relative increases observed upon the deletion of the *5´UTR*$_{B7}$, it appears that repression mediated by the *5´UTR*$_{B7}$ leads to a reduction of VirB protein production by, on average 3.8-fold (± 0.4) over all conditions tested for both strains (Figure 4A, fold-changes are indicated above each pair of boxplots). As such, it seems that CsrA represses the *virB* operon to an extent that is largely independent of the carbohydrate source or casamino acid availability.

Homeostasis of X-T4SS density in the cell envelope over a wide range of nutrient availability

Spurred by the increased expression of the *virB* operon during decreased inputs of casamino acids, we set out to assess the number of assembled T4SS over a wide range of casamino acid and sucrose concentrations. For this, an 8 by 8 matrix of wells containing defined media with varying nutrient levels was created in a 96-well plate. With sucrose (rows) and casamino acids (columns) ranging from 0.4% to 0.007% in a 1.8x dilution series. After 24 hours of growth and an additional 5 hours of growth after a 4-fold dilution in the same but fresh media, *X. citri virB10-msfgfp*$_{TL}$ cells were sampled and immediately imaged by fluorescence microscopy, registering both cell dimensions and the number of T4SS foci present per cell. In total, 42 different conditions (Figure 4B inset) were sampled over two separate experiments). Figures 4B and 4C show that the different nutritional inputs in each well result in a range of cell sizes, represented by their average surface areas and reveal a general correlation of reduced cell size with reduced casamino acid or sucrose availability.

Importantly, plotting the number of T4SSs versus cellular surface area shows a linear increase in T4SS numbers with increasing surface area (Figure 4D; Pearson correlation r = 0.34, as calculated for all data points). Furthermore, this positive correlation in turn reflects an almost constant average T4SS density (T4SS/surface area) in the cell envelope with a subtle increase observed for the smallest cells (Figure 4D inset). This is in line with the observation of subtly increased msfGFP production in the *X. citri virB11-msfGFP* transcriptional reporter at low casamino acid concentrations (Figure 4A). These results suggest that during the cell-cycle, when the surface area gradually increases until cell division, T4SSs are added continuously. Therefore, it seems that an *X. citri* population maintains the density of its T4SSs within a specific range under a variety of nutritional conditions. The number of T4SSs relative to surface area (T4SS density) could be an important factor in determining the probability that an *X. citri* cell is able to successfully transfer effectors into a neighbouring target cell during interbacterial competition.

X-T4SS overproduction in a $\Delta 5'UTR_{B7}$ background has an impact on X. citri physiology and leads to reduced growth speeds

Since *$\Delta 5'UTR_{B7}$* cells present a roughly 4-fold greater expression from the *virB* operon (Figure 4A) and kills with approximately twice the efficiency as wild-type cells (Fig. 3B), we asked whether this potentially advantageous feature could be counter-balanced by the inherent metabolic cost of T4SS production. We therefore set up a co-culture experiment to test whether overproduction of T4SSs in the *$\Delta 5'UTR_{B7}$* background leads to a detectable growth disadvantage in *X. citri*. For this, we took advantage of the difference in msfGFP production levels between *X. citri virB11-msfgfp* and *X. citri $\Delta 5'UTR_{B7}$ virB11-msfgfp* (Figures 1E and 4A) to sort wild-type and overproducing *$\Delta 5'UTR_{B7}$* cells by fluorescence microscopy. This mitigated the need to introduce different antibiotic resistance markers in the genome that could on their own lead to subtle physiological differences. Additionally, the strains used here are genetically very closely related since they were obtained from the same recombination events leading to either the wild-type or mutant *$5'UTR_{B7}$* allele (see Materials and Methods). Separate cultures of different single colonies of each of the strains were grown in defined AB media before being mixed and diluted in a 1:1 ratio into AB media supplemented with 0.2% sucrose and 0.01% casamino acids. Cultures were diluted regularly to avoid saturation and fluorescence microscopy images of thousands of cells were obtained

at different time points over a period of approximately one week. Figure 5A shows the results of two distinct experiments, using a total of seven separate cultures, where we observed a 19% and 13% average decrease in the *X. citri* $\Delta 5'UTR_{B7}$ *virB11-msfgfp* cell population relative to the wild-type *X. citri virB11-msfgfp* cell population. Additionally, analysis of the cell sizes of the two strains at several time points over a one-week period revealed that cells with the $\Delta 5'UTR_{B7}$ background consistently have a slightly larger surface area over volume ratio (SA/V; Figure 5B), indicative of smaller cell sizes. The SA/V ratio has been suggested to be a measure of the physiological state of rod-shaped bacterial cells and is thought to be set by the availability of nutrients (Harris and Theriot, 2016, 2018). The observed average size reduction (increase in SA/V) for *X. citri* $\Delta 5'UTR_{B7}$ *virB11-msfgfp* cells could be attributed to the increased metabolic cost of nutrients consumed in T4SS production and/or to stress induced by the greater number of T4SSs in the cell envelope. This experiment reveals that T4SS production has a measurable cost and illustrates the importance of balancing expenses with gains from increased aggressiveness during interbacterial competition.

Discussion

We show here that the regulation of the *virB* operon in *X. citri*, coding for the structural proteins of its bacteria-killing X-T4SS, is under control of the global regulator CsrA which binds to sites in the 5´ untranslated region of the polycistronic mRNA initiating at the *virB7* gene (Figure 1). The removal of the $5'UTR_{B7}$ leads to an increase in production of *virB* encoded proteins, which in turn leads to an increase in number of T4SSs present in the cell envelope and greater interbacterial killing efficiency (Figures 1E, 3A and 3B). Despite CsrA repression over the *virB* operon, we have not yet found any growth condition that would greatly decrease nor increase T4SS production and T4SS-dependent killing by *X. citri* cells (Figures 3C, 3D and 4A). Therefore, the removal of CsrA repression does not seem to be required to induce production of the T4SS. Rather, CsrA repression maintains a discrete number of T4SSs in the cell envelope over a range of different growth conditions tested (Figures 3 and 4).

Several factors can be imagined to be influencing the efficiency with which an *X. citri* cell can mount a successful contact-dependent attack. Firstly, an X-T4SS needs to be present

at the contact interface between the attacking *X. citri* cell and the target rival cell. After a successful contact, X-T4SS effectors need to be translocated through the X-T4SS. This is dependent on both the availability of effectors and, importantly, ATP to power the secretion. For example, the subtle decrease in killing efficiencies in the sucrose-depleted conditions (Figure 3D) might stem from reduced energy levels, since sucrose would be the main carbohydrate fed into the glycolysis and citric acid metabolic pathways. Furthermore, depleting nutrients also leads to much smaller cell sizes compared to the cells grown in rich media (Harris and Theriot, 2016). This reduction in cell size in itself could influence killing-efficiencies by increasing the probability of small cells contacting larger *E. coli* cells when in co-culture on a solid surface, since smaller *X. citri* cells will have a more close-packed arrangement next to *E. coli* cells (Hudson, 1949), increasing the probabilities of successful T4SS contact. Expanding this line of reasoning, a unit mass of cells formed during growth in reduced nutrient conditions would have a higher contact probability as the same mass of cells formed during growth in rich conditions, because of the former's greater number of single cells and greater SA/V ratio for each individual cell. Thus, the subtle increase in T4SS density with lower nutrient inputs (Figure 4B) and the concomitant smaller cell sizes, could both be responsible for the slight increments in killing efficiency during growth in media depleted in both casamino acids and sucrose (Figure 3D).

The complex regulatory features governing CsrA production and activity, including negative autoregulation, have been proposed to act to keep CsrA levels and activity relatively stable with greatly reduced cell-to-cell variability, making CsrA an ideal regulator for homeostatic responses (Romeo and Babitzke, 2018; Yakhnin et al., 2011). Of note, an ancestral CsrA homolog has been shown to act as a homeostatic control agent during flagella morphogenesis in the Gram-positive *Bacillus subtilis* (Mukherjee et al., 2011). As such, CsrA could integrate diverse signals that relay information regarding the nutritional environment of the cell and subsequently stabilise its own activity to ensure a constant regulatory effect on its target transcripts. In light of this model for CsrA function, our experiments do indeed indicate that in *X. citri*, this protein acts to reduce production from the *virB* operon with roughly the same repressing power over a wide range of growth conditions (Figure 4A). Recently, it was reported that CsrA (RsmA) represses all three type VI secretion systems from *P. aeruginosa*, abolishing translation under non-inducing conditions (Allsopp et al., 2017). Affinity of RNA loops for CsrA can differ by several orders of

magnitude (Duss et al., 2014) and so some CsrA-mRNA associations will be very sensitive to fluctuations in mRNA levels and CsrA availability while others remain insensitive. As such, CsrA-mRNA affinities could be tuned so as to constitutively stabilise translation from one transcript at basal levels (such as for the *virB* operon) and at the same time ensure that translation from other transcripts is only derepressed by a specific trigger; for example, by a specific condition that changes the mRNA structure. Several genetic circuits leading to different outcomes are possible, but for sake of general discussion, we note that it is unlikely that CsrA availability varies greatly under different conditions, since this would be hard to reconcile with the simultaneous control of the hundreds of transcripts through which CsrA exerts its pleiotropic effects (Andrade et al., 2014; Potts et al., 2017).

The removal of CsrA-based repression of T4SS production results in a two-fold increase in interspecies bacteria killing efficiency (Figure 3B) which could be beneficial under certain circumstances. However, the production of a T4SS has its costs: the maintenance and transcription of an approximately 13 kb locus, multiple rounds of translation to produce the over 100 subunits that need to be transported to the cell envelope and assembled into a single system of over 3 MDa in size (Low et al., 2014). Since the maintenance of even a single gene and the production of the amino acids to build up its protein product has a measurable cost (Akashi and Gojobori, 2002; Lynch and Marinov, 2015; Wagner, 2005), it was not surprising that we observed a reduction in the fitness of the *X. citri* strain in which CsrA-based repression was removed (Figure 5).

Interbacterial competition is increasingly being shown to be crucial for fitness, survival (García-Bayona and Comstock, 2018; Lories et al., 2017) and structuring of bacterial populations (Nadell et al., 2016) and possibly contribute to bacterial evolution by uptake of DNA from lysed target cells (Veening and Blokesch, 2017). The continuous expression and activity of the X-T4SS under different growth conditions, further illustrates the importance of interbacterial killing and the benefits that are accompanied with it. The control of *X. citri* X-T4SS production by CsrA seems to guarantee constant densities of this nanomachine at the bacterial surface, high enough to provide protection against rival bacteria, but not so high as to represent a metabolic burden. In this way, a balance is maintained that maximizes long-term *X. citri* survival in the varied natural environments it encounters during its life cycle, both within and outside of its plant host.

Materials and Methods

Bacterial strains, media and culturing

All strains used are listed in Supplementary Table 1. For all experiments, strains were grown in defined AB media containing 15mM $(NH_4)_2SO_4$, 17mM Na_2HPO_4, 22mM KH_2PO_4, 50mM NaCl, 0.1 mM $CaCl_2$, 1 mM $MgCl_2$ and 3 µM $FeCl_3$, at pH 7.0, supplemented with 10 µg/mL thiamine and 25 µg/mL uracil and varying concentrations of carbohydrate sources and casamino acids as described in the text. For cloning purposes standard lysogeny broth (5g/l yeast extract, 5 g/l NaCl, 10 g/l tryptone and 15 g/l agar) and 2xYT (5 g/l yeast extract, 10 g/l NaCl, 16 g/l tryptone and 15 g/l agar) were used. For counterselection, sucrose plates were used (5 g/l yeast extract, 10 g/l tryptone, 60 g/l sucrose). Standard incubations were performed at 28°C in 24-well plates using 1.5 mL culture media or in 96-well plates with 200 µL culture media with shaking at 200 rpm. In general, after a first overnight growth period in 2xYT medium, cells were transferred at a 100-fold dilution into AB defined media for a second overnight growth to synchronise growth. Cells were then diluted in fresh media and, after 4- to 6-hour growth, imaged by microscopy. In case of experiments involving different growth media compositions, cultures were inoculated once more at a 100-fold dilution in the appropriate AB media composition for overnight growth and a final re-inoculation in fresh media with dilutions ranging from 2-fold to 100-fold, depending on the overnight attained optical densities. Note that cultures grown under different nutrient conditions attained different densities after overnight growth. Care was therefore taken to dilute each culture in its appropriate media such as to obtain adequate numbers of cells for experimental assays but at the same time avoiding saturation of the faster growing cultures. After a final 5- to 9-hour growth period, cells were either imaged with fluorescence microscopy or subjected to competition assays.

Cloning of constructs for genomic insertions and deletions

All primers, plasmids and strains used for cloning and PCR verifications together with brief description of the constructions are listed in Supplementary Table 1. Genomic deletions

and insertions in the *X. citri* genome were all constructed using a two-step allelic exchange procedure (Hmelo et al., 2015). For this, 500 to 1000 base pair-sized fragments up- and downstream from the region of interest were amplified using a high-fidelity polymerase (Phusion, Thermo Scientific) and cloned into the pNPTS138 vector either by traditional restriction digest cloning (NEB and Thermo Scientific) or by Gibson assembly (NEB). The resulting plasmid was used to transform the appropriate *X. citri* strain by electroporation (2.0 kV, 200 Ω, 25 µF, 0.2 cm cuvettes; Bio-Rad)(Sawitzke et al., 2011). A first recombination event was selected for on LB plates containing 50 µg/ml kanamycin. Transformants were streaked for single colonies on kanamycin plates whereafter several single colonies of the merodiploids (Kan^R, Suc^S) were streaked on sucrose plates selecting for a second recombination event creating either a wild-type (reversion) or mutant allele. After confirmation of the loss of the kanamycin resistance cassette together with *sacB*, a PCR was performed using primers that hybridize outside of the homology regions to identify the target allele. Strains containing the wild-type alleles (revertants created at an equal rate during the second recombination event) were also stored and used as controls for their respective mutants. For the insertion of the pPM7G plasmid into the *amy* gene of *X. citri,* cells were electroporated with the pPM7G plasmid and selected for on LB plates containing 50 µg/mL kanamycin. Integrity of the *virB* operon in these strains was confirmed by PCR to exclude erroneous recombination with the pPM7G cloned $5´UTR_{B7}$ regions.

Chlorophenol red-β-D-galactopyranoside (CPRG) bacterial competition assay

To visualize and quantify the ability of *X. citri* to lyse *E. coli* strain MG1655, a CPRG-based method was used as described previously (Sgro et al., 2018; Vettiger and Basler, 2016). Briefly, to each well of a clear U-shaped bottom 96-well plate, 100 µL of a mixture of 0.5X buffer A (7.5mM $(NH_4)_2SO_4$, 8.5mM Na_2HPO_4, 11mM KH_2PO_4, 25mM NaCl, pH 7.0), 1.5% agarose and 40 µg/mL CPRG (Sigma-Aldrich) was added, and plates were thoroughly dried under a laminar flow. *X. citri* cells grown in the appropriate media, were mixed in a 1:1 volume ratio with a concentrated *E. coli* culture. The *E. coli* cultures were grown to OD_{600} = 1 in the presence of 0.2 mM IPTG (inducing the *lac* operon) in 2xYT medium, washed once and concentrated 10 times. Five microliters of *X. citri* and *E. coli* mixtures were immediately added to the 96-well plate without puncturing or damaging the agarose, covered with a

transparent seal and quickly thereafter absorbance at 572 nm (A_{572}) was monitored over time in a 96-well plate reader for at least 200 minutes (SpectraMax Paradigm, Molecular Devices). The A_{572} values were processed using RStudio software (RStudio-Team, 2016) and plotted using the ggplot2 package (Wickham, 2016). Background intensities obtained from the mean of A_{572} values of unchallenged, non-lysing *E. coli* cells were subtracted from the data series and data were normalized for initial OD_{600} differences.

Fluorescence microscopy image acquisition and analysis

Briefly, 1 µL of cell suspension was spotted on a thin agarose slab containing 1X buffer A (15mM $(NH_4)_2SO_4$, 17mM Na_2HPO_4, 22mM KH_2PO_4, 50mM NaCl, pH 7) and 2% agarose and covered with a #1.5 cover glass (Corning). For time-lapse imaging, thicker agar slabs containing the appropriate media were constructed as described (Bayer-Santos et al., 2019). Phase contrast and msfGFP emission images were obtained with a Leica DMi-8 epifluorescent microscope. msfGFP emissions were captured using 1000 to 1500 ms exposure times at maximum excitation light intensities. The microscope was equipped with a DFC365 FX camera (Leica), a HC PL APO 100x/1.4 Oil ph3 objective (Leica) and excitation-emission band-pass filter cubes for GFP (Ex.: 470/40, DC: 495, Em.: 525/50; Leica) foci, eleven 0.05 µm Z-plane stacks were obtained from a 0.5 µm region within the centre of the cells. This allowed for a better signal to noise ratio and increased detection of foci location in different depths of the cell. These image stacks were background subtracted by a rolling ball correction using a significant cell-free portion of each image as a reference and, finally, combined by an average intensity projection using the FIJI software package (Schindelin et al., 2012). To obtain a quantitative representation of cell sizes, background corrected fluorescence intensities and amount of foci present per cell, the images were analysed using the MicrobeJ software package (Ducret et al., 2016) and data was analysed by RStudio software (RStudio-Team, 2016) and plotted using the ggplot2 package (Wickham, 2016).

Co-culture growth experiment

To illustrate the physiological burden associated with T4SS overexpression seven independent *X. citri* $\Delta 5'UTR_{B7}$ *virB11-msfgp* mutants and seven independent *X. citri virB11-*

msfgfp strains (revertants to wild-type from the second recombination event), in two separate experiments, were grown overnight in 2xYT media, diluted 100-fold into defined AB media with 0.2% sucrose and 0.2% casamino acids and grown overnight. These overnight cultures were mixed 1:1 and 10-fold diluted into fresh AB media with 0.2% sucrose and 0.01% casamino acids. Immediately after mixing (at time point 0 h) fluorescence microscopy images were taken (as described above) to register the exact ratio of *X. citri virB11-msfgfp* cells versus *X. citri* $\Delta 5'UTR_{B7}$ *virB11-msfgfp* cells. The cultures were subsequently diluted regularly so to prevent cultures from reaching saturation which would halt further cell division. At the indicated time-points several microscopy images of each of the co-cultures were again acquired. Given that the *virB11-msfgfp* reporter in the strain lacking the $5'UTR_{B7}$ has a higher msfGFP production (histograms of the populations' msfGFP fluorescence levels do not overlap), cells could be sorted by using average msfGFP content and as such, an accurate quantification of the cell ratio between wild-type and deleted $5'UTR_{B7}$ strains could be calculated over time.

Transcription start site analysis

Transcriptional start sites of the *virD* and *virB* transcripts were identified using the 5´ RACE Kit (Roche) as described (Andrade et al., 2014). The oligonucleotide sequences for *virD4* and *virB7* 5´ RACE assays are listed in Supplementary Table 1. The resulting PCR fragments were blunt ligated into pGEM-T before sequencing 3 independent clones.

RNA electrophoretic mobility shift assays

DNA fragments encoding either the entire 5´ UTR of the *virB* operon or a shortened fragment lacking the first 73 nucleotides (Δnt1-73; Figure 1), were amplified from *X. citri* genome using forward primers which include the T7 promoter sequence (see Supplementary Table 1). RNA transcripts of the cloned $5'UTR_{B7}$ fragments were produced *in vitro* from the resulting purified PCR products by using the T7 Transcription Kit (Roche) and labelled by using the RNA 3´ End Biotinylation Kit (Pierce). Recombinant CsrA protein was purified as previously described (Andrade et al., 2014). Approximately 70 nM of purified CsrA protein and 6.25 nM Biotin-labeled RNA were mixed with binding buffer (10 mM HEPES pH 7.3, 20 mM KCl, 1mM MgCl2, 1 mM DTT, 5% glycerol, 0.1 µg/µL yeast tRNA, 20 U RNasin

(Promega)) in a total reaction volume of 20 µL. The binding reactions were incubated at 25°C for 20 min. A 5 µL aliquot of loading buffer (97% glycerol, 0.01% bromophenol blue, 0.01% xylene cyanol) was added to the binding reaction and immediately loaded and resolved by 5% native polyacrylamide gels. The binding assays and detection of RNA products were performed with the LightShift Chemiluminescent RNA EMSA Kit (Thermo Scientific). For the control reactions, 312.5 nM unlabelled (competitor) *virB 5´UTR*$_{B7}$ RNA was added to the binding reactions.

Western blot assays

Western blot assays were performed using total protein extract of wild-type and mutant *X. citri* strains as described previously (Sgro et al., 2018). Specific polyclonal antibodies against VirB7, VirB8, VirB9, VirB10 and X-Tfe^{XAC2609} were used. In order to correct for sample loading variations, levels of the β subunit of RNA polymerase (RNApolβ) were also evaluated. Briefly, *X. citri* cells grown overnight in AB medium supplemented with 0.2% sucrose and 0.5% casamino acids were, collected by centrifugation, resuspended, and quickly adjusted to the same optical density (OD_{600nm}=1.3). Cells from 1 mL were harvested by centrifugation for 5 min at 6000g, resuspended in 500 µL of 1X PBS plus 125 µL of denaturing sample buffer and incubated for 5 min at 95°C. Samples were resolved in 15-well Tricine-SDS-PAGE gels (Schagger, 2006), transferred to 0.2 µm nitrocellulose membranes (Bio Rad), and blocked for 60 min using 5% skimmed milk in 1XPBS. Primary antibodies produced in rabbit against VirB7 (AbVirB7; 1:20,000 dilution), VirB8 (AbVirB8; 1:10,000), VirB9 (AbVirB9; 1:4,000), VirB10 (AbVirB10; 1:5,000), X-Tfe^{XAC2609} (AbXAC2609; 1:4,000) and in mouse against the β-subunit of RNA polymerase from *E. coli* (AbRNApolβ; Abcam ab12087; 1:5,000) were used. Secondary goat anti-rabbit or anti-mouse IgG-AP conjugates (Bio Rad 1706518; 1:5,000) were used for AbVirB7, AbVirB8, AbXAC2609 and AbRNApolβ with BCIP (VWR 0885) and NBT (Sigma-Aldrich N6876) for protein detection, and secondary goat anti-rabbit IgG-IRDye 800CW (Li-Cor 32211; 1:8,000) was used for AbVirB9 and AbVirB10 before visualization using an Odyssey Imaging System (Li-Cor). Band intensities were quantified using the FIJI software package (Schindelin et al., 2012) and normalized with respect to the intensities obtained in the *X. citri* wild-type strain for each protein.

Results represent the mean value from at least four experiments (minimum two biological replicates) and vertical lines the corresponding standard deviation.

RNA and cDNA preparation

Bacterial total RNA was isolated from *X. citri* strains (wild-type and derivatives) using either TRIzol® reagent (Thermo Scientific, 15596026) or RNAspin Mini Kit (GE, 25-0500-71) according to the manufacturer´s instructions. Approximately 10^8 cells from an overnight culture in AB medium were used for each extraction. For cDNA first strand synthesis, 4 µg of total RNA were reverse transcribed using RevertAid First Strand cDNA Synthesis Kit (Thermo Scientific, K1622), according to the manufacturer's instructions and cDNA concentrations were subsequently adjusted to 0.1 µg/µL. The absence of contamination by genomic DNA in each sample was confirmed by means of PCR analysis using 16S-specific oligonucleotides.

RT-qPCR and overlapping PCR assays

Real-time quantitative polymerase chain reaction (RT-qPCR) assays were performed using Maxima SYBR Green/ROX qPCR Master Mix (Thermo Scientific, K0222) according to manufacturer's instructions in a QuantStudio 3 Real-Time PCR System (Applied Biosystems). The oligonucleotide sequences used to analyse the expression of each gene can be found in Supplementary Table 1. Results were analysed with the QuantStudio Design and Analysis Software (Applied Biosystems) and processed using the ΔΔCt method as described (Livak and Schmittgen, 2001) using the 16S gene as the internal reference and *X. citri* wild-type strain as the reference sample. Plotted values represent the mean from four RT-qPCR runs (two biological and two technical replicates for each gene), and vertical lines the accumulated error from standard deviations. Overlapping PCR assays were performed using Phusion High-Fidelity PCR Master Mix (Thermo Scientific, F531L) according to manufacturer's instructions. Nine pairs of oligonucleotides (named from I to IX) were employed (see Supplementary Table 1). The following templates were used: non-reverse transcribed *X. citri* RNA (negative amplification control), reverse transcribed *X. citri* RNA obtained as described above (sample) and *X. citri* genomic DNA (positive amplification control). All PCR products were resolved in SYBR Safe (Invitrogen, S33102) stained 1%

agarose gels. Overlapping PCR assays were performed in two biological replicates with similar results.

Acknowledgments

We thank Alexandre Bruni-Cardoso for unlimited access to the Leica DMI8 microscope, Ioannis Passaris and Sander K. Govers for helpful discussions, suggestions and critical reading of the manuscript, Gilberto Kaihami for the introduction to the R programming language. This work was supported by FAPESP post-doctoral scholarships to William Cenens (2015/18237-2), Germán G. Sgro (2014/04294-1), and Edgar Llontop (2019/12234-2) and FAPESP research grants to Chuck S. Farah (2011/07777-5 and 2017/17303-7).

References

Abendroth, U., Schmidtke, C., and Bonas, U. (2014). Small non-coding RNAs in plant-pathogenic Xanthomonas spp. RNA Biol *11*, 457–463.

Akashi, H., and Gojobori, T. (2002). Metabolic efficiency and amino acid composition in the proteomes of Escherichia coli and Bacillus subtilis. Proc. Natl. Acad. Sci. *99*, 3695–3700.

Alkhateeb, R.S., Vorhölter, F.-J., Rückert, C., Mentz, A., Wibberg, D., Hublik, G., Niehaus, K., and Pühler, A. (2016). Genome wide transcription start sites analysis of Xanthomonas campestris pv. campestris B100 with insights into the gum gene cluster directing the biosynthesis of the exopolysaccharide xanthan. J. Biotechnol. *225*, 18–28.

Allsopp, L.P., Wood, T.E., Howard, S.A., Maggiorelli, F., Nolan, L.M., Wettstadt, S., and Filloux, A. (2017). RsmA and AmrZ orchestrate the assembly of all three type VI secretion systems in Pseudomonas aeruginosa. Proc. Natl. Acad. Sci. U. S. A. *114*, 7707–7712.

Andrade, M.O., Farah, C.S., and Wang, N. (2014). The Post-transcriptional Regulator rsmA/csrA Activates T3SS by Stabilizing the 5′ UTR of hrpG, the Master Regulator of hrp/hrc Genes, in Xanthomonas. PLoS Pathog. *10*.

Baker, C.S., Morozov, I., Suzuki, K., Romeo, T., and Babitzke, P. (2002). CsrA regulates

glycogen biosynthesis by preventing translation of glgC in Escherichia coli. Mol. Microbiol. *44*, 1599–1610.

Bayer-Santos, E., Lima, L. dos P., Ceseti, L. de M., Ratagami, C.Y., de Santana, E.S., da Silva, A.M., Farah, C.S., and Alvarez-Martinez, C.E. (2018). Xanthomonas citri T6SS mediates resistance to Dictyostelium predation and is regulated by an ECF σ factor and cognate Ser/Thr kinase. Environ. Microbiol.

Bayer-Santos, E., Cenens, W., Matsuyama, B.Y., Oka, G.U., Di Sessa, G., Del Valle Mininel, I., Alves, T.L., and Farah, C.S. (2019). The opportunistic pathogen Stenotrophomonas maltophilia utilizes a type IV secretion system for interbacterial killing. PLoS Pathog.

Borgeaud, S., Metzger, L.C., Scrignari, T., and Blokesch, M. (2015). The type VI secretion system of Vibrio cholerae fosters horizontal gene transfer. Science (80-.). *347*, 63–67.

Boschiroli, M.L., Ouahrani-Bettache, S., Foulongne, V., Michaux-Charachon, S., Bourg, G., Allardet-Servent, A., Cazevieille, C., Liautard, J.P., Ramuz, M., and O'Callaghan, D. (2002). The Brucella suis virB operon is induced intracellularly in macrophages. Proc. Natl. Acad. Sci. *99*, 1544–1549.

Chao, N.-X., Wei, K., Chen, Q., Meng, Q.-L., Tang, D.-J., He, Y.-Q., Lu, G.-T., Jiang, B.-L., Liang, X.-X., Feng, J.-X., et al. (2008). The rsmA -like Gene rsmA Xcc of Xanthomonas campestris pv. campestris Is Involved in the Control of Various Cellular Processes, Including Pathogenesis . Mol. Plant-Microbe Interact. *21*, 411–423.

Chung, J.M., Sheedlo, M.J., Campbell, A.M., Sawhney, N., Cheng, A.E.F., Lacy, D.B., Cover, T.L., and Ohi, M.D. (2019). Structure of the helicobacter pylori cag type IV secretion system. Elife.

Das, A., and Pazour, P.J. (1989). Delineation of the regulatory region sequences of Agrobacterium tumefaciens virB operon. Nucleic Acids Res. *17*, 4541–4550.

Dehio, C., and Tsolis, R.M. (2017). Type IV effector secretion and subversion of host functions by bartonella and brucella species. In Current Topics in Microbiology and Immunology, (Springer, Cham), pp. 269–295.

Ducret, A., Quardokus, E.M., and Brun, Y. V. (2016). MicrobeJ, a tool for high throughput bacterial cell detection and quantitative analysis. Nat. Microbiol. *1*, 16077.

Duss, O., Michel, E., Dit Konté, N.D., Schubert, M., and Allain, F.H.T. (2014). Molecular basis for the wide range of affinity found in Csr/Rsm protein-RNA recognition. Nucleic Acids Res. *42*, 5332–5346.

Gao, R., and Lynn, D.G. (2005). Environmental pH sensing: Resolving the VirA/VirG two-component system inputs for Agrobacterium pathogenesis. J. Bacteriol. *187*, 2182–2189.

García-Bayona, L., and Comstock, L.E. (2018). Bacterial antagonism in host-associated microbial communities. Science (80-.). *361*, eaat2456.

Ghosal, D., Jeong, K.C., Chang, Y.W., Gyore, J., Teng, L., Gardner, A., Vogel, J.P., and Jensen, G.J. (2019). Molecular architecture, polar targeting and biogenesis of the Legionella Dot/Icm T4SS. Nat. Microbiol.

Gordon, J.E., and Christie, P.J. (2014). The Agrobacterium Ti Plasmids. Microbiol. Spectr. *2*, 1–18.

Harris, L.K., and Theriot, J.A. (2016). Relative Rates of Surface and Volume Synthesis Set Bacterial Cell Size. Cell *165*, 1479–1492.

Harris, L.K., and Theriot, J.A. (2018). Surface Area to Volume Ratio: A Natural Variable for Bacterial Morphogenesis. Trends Microbiol. *26*, 815–832.

Hmelo, L.R., Borlee, B.R., Almblad, H., Love, M.E., Randall, T.E., Tseng, B.S., Lin, C., Irie, Y., Storek, K.M., Yang, J.J., et al. (2015). Precision-engineering the Pseudomonas aeruginosa genome with two-step allelic exchange. Nat. Protoc. *10*, 1820–1841.

Holmqvist, E., Wright, P.R., Li, L., Bischler, T., Barquist, L., Reinhardt, R., Backofen, R., and Vogel, J. (2016). Global RNA recognition patterns of post-transcriptional regulators Hfq and CsrA revealed by UV crosslinking in vivo. EMBO J. *35*, e201593360.

Hu, B., Khara, P., and Christie, P.J. (2019). Structural bases for F plasmid conjugation and F pilus biogenesis in Escherichia coli. Proc. Natl. Acad. Sci. U. S. A.

Hudson, D.R. (1949). Density and Packing in an Aggregate of Mixed Spheres. J. Appl. Phys. *20*, 154–162.

Ilangovan, A., Connery, S., and Waksman, G. (2015). Structural biology of the Gram-negative bacterial conjugation systems. Trends Microbiol. *23*, 301–310.

Janssen, K.H., Diaz, M.R., Golden, M., Graham, J.W., Sanders, W., Wolfgang, M.C., and Yahr, T.L. (2018). Functional analyses of the RsmY and RsmZ small noncoding regulatory RNAs in Pseudomonas aeruginosa.

Koraimann, G., and Wagner, M.A. (2014). Social behavior and decision making in bacterial conjugation. Front. Cell. Infect. Microbiol. *4*, 1–7.

Liu, M.Y., and Romeo, T. (1997). The global regulator CsrA of Escherichia coli is a specific mRNA-binding protein. J. Bacteriol. *179*, 4639–4642.

Livak, K.J., and Schmittgen, T.D. (2001). Analysis of relative gene expression data using real-time quantitative PCR and the 2(-Delta Delta C(T)) Method. Methods *25*, 402–408.

Lohrke, S.M., Yang, H., and Jin, S. (2001). Reconstitution of Acetosyringone-mediated Agrobacterium tumefaciens virulence gene expression in the heterologous host Escherichia coli. J. Bacteriol. *183*, 3704–3711.

Lories, B., Parijs, I., Foster, K.R., and Steenackers, H.P. (2017). Meeting report on the ASM Conference on Mechanisms of Interbacterial Cooperation and Competition. J. Bacteriol. JB.00403-17.

Low, H.H., Gubellini, F., Rivera-Calzada, A., Braun, N., Connery, S., Dujeancourt, A., Lu, F., Redzej, A., Fronzes, R., Orlova, E. V, et al. (2014). Structure of a type IV secretion system. Nature *508*, 550–553.

Lu, X.H., An, S.Q., Tang, D.J., McCarthy, Y., Tang, J.L., Dow, J.M., and Ryan, R.P. (2012). RsmA Regulates Biofilm Formation in Xanthomonas campestris through a Regulatory Network Involving Cyclic di-GMP and the Clp Transcription Factor. PLoS One *7*, 1–11.

Lynch, M., and Marinov, G.K. (2015). The bioenergetic costs of a gene. Proc. Natl. Acad. Sci. *112*, 201514974.

Mansfield, J., Genin, S., Magori, S., Citovsky, V., Sriariyanum, M., Ronald, P., Dow, M., Verdier, V., Beer, S. V, Machado, M.A., et al. (2012). Top 10 plant pathogenic bacteria in molecular plant pathology. Mol Plant Pathol *13*, 614–629.

Marteyn, B., West, N.P., Browning, D.F., Cole, J.A., Shaw, J.G., Palm, F., Mounier, J., Prévost, M.C., Sansonetti, P., and Tang, C.M. (2010). Modulation of Shigella virulence in response to available oxygen in vivo. Nature *465*, 355–358.

Moumène, A., Gonzalez-Rizzo, S., Lefrançois, T., Vachiéry, N., and Meyer, D.F. (2017). Iron Starvation Conditions Upregulate Ehrlichia ruminantium Type IV Secretion System, tr1 Transcription Factor and map1 Genes Family through the Master Regulatory Protein ErxR. Front. Cell. Infect. Microbiol. *7*, 535.

Mukherjee, S., Yakhnin, H., Kysela, D., Sokoloski, J., Babitzke, P., and Kearns, D.B. (2011). CsrA-FliW interaction governs flagellin homeostasis and a checkpoint on flagellar morphogenesis in Bacillus subtilis. Mol. Microbiol. *82*, 447–461.

Nadell, C.D., Drescher, K., and Foster, K.R. (2016). Spatial structure, cooperation and competition in biofilms. Nat. Rev. Microbiol. *14*, 589–600.

Potts, A.H., Vakulskas, C.A., Pannuri, A., Yakhnin, H., Babitzke, P., and Romeo, T. (2017).

Global role of the bacterial post-transcriptional regulator CsrA revealed by integrated transcriptomics. Nat. Commun. *8*, 1596.

Romeo, T., and Babitzke, P. (2018). Global Regulation by CsrA and Its RNA Antagonists. Microbiol. Spectr. *6*, 1–14.

RStudio-Team (2016). RStudio: Integrated Development for R.

Sawitzke, J.A., Costantino, N., Li, X.T., Thomason, L.C., Bubunenko, M., Court, C., and Court, D.L. (2011). Probing cellular processes with oligo-mediated recombination and using the knowledge gained to optimize recombineering. J. Mol. Biol. *407*, 45–59.

Schagger, H. (2006). Tricine-SDS-PAGE. Nat Protoc *1*, 16–22.

Schindelin, J., Arganda-Carreras, I., Frise, E., Kaynig, V., Longair, M., Pietzsch, T., Preibisch, S., Rueden, C., Saalfeld, S., Schmid, B., et al. (2012). Fiji: An open-source platform for biological-image analysis. Nat. Methods *9*, 676–682.

Schulte, R., and Bonas, U. (1992). Expression of the Xanthomonas campestris pv. vesicatoria hrp gene cluster, which determines pathogenicity and hypersensitivity on pepper and tomato, is plant inducible. J Bacteriol *174*, 815–823.

Sgro, G.G., Costa, T.R.D., Cenens, W., Souza, D.P., Cassago, A., Coutinho de Oliveira, L., Salinas, R.K., Portugal, R. V., Farah, C.S., and Waksman, G. (2018). Cryo-EM structure of the bacteria-killing type IV secretion system core complex from Xanthomonas citri. Nat. Microbiol.

Sgro, G.G., Oka, G.U., Souza, D.P., Cenens, W., Bayer-Santos, E., Matsuyama, B., Bueno, N.F., Dos Santos, T.R., Alvarez-Martinez, C.E., Salinas, R.K., et al. (2019). Bacteria-killing Type IV Secretion Systems. Front. Microbiol. *10*, 1078.

Souza, D.P., Oka, G.U., Alvarez-Martinez, C.E., Bisson-Filho, A.W., Dunger, G., Hobeika, L., Cavalcante, N.S., Alegria, M.C., Barbosa, L.R.S., Salinas, R.K., et al. (2015). Bacterial killing via a type IV secretion system. Nat. Commun. *6*, 1–9.

Veening, J.-W., and Blokesch, M. (2017). Interbacterial predation as a strategy for DNA acquisition in naturally competent bacteria. Nat. Rev. Microbiol. *15*, 621–629.

Vergunst, A.C., Schrammeijer, B., den Dulk-Ras, A., de Vlaam, C.M.T., Regensburg-Tuink, T.J.G., and Hooykaas, P.J.J. (2000). VirB / D4-Dependent Protein Translocation from Agrobacterium into Plant Cells. Science (80-.). *290*, 979–982.

Vettiger, A., and Basler, M. (2016). Type VI Secretion System Substrates Are Transferred and Reused among Sister Cells. Cell *167*, 99-110.e12.

Wagner, A. (2005). Energy constraints on the evolution of gene expression. Mol. Biol. Evol. *22*, 1365–1374.

Weilbacher, T., Suzuki, K., Dubey, A.K., Wang, X., Gudapaty, S., Morozov, I., Baker, C.S., Georgellis, D., Babitzke, P., and Romeo, T. (2003). A novel sRNA component of the carbon storage regulatory system of Escherichia coli. Mol. Microbiol. *48*, 657–670.

Wickham, H. (2016). ggplot2: Elegant Graphics for Data Analysis (Springer-Verlag New York).

Winans, S.C. (1990). Transcriptional induction of an Agrobacterium regulatory gene at tandem promoters by plant-released phenolic compounds, phosphate starvation, and acidic growth media. J. Bacteriol. *172*, 2433–2438.

Yakhnin, H., Yakhnin, A. V, Baker, C.S., Sineva, E., Berezin, I., Romeo, T., and Babitzke, P. (2011). Complex regulation of the global regulatory gene csrA: CsrA-mediated translational repression, transcription from five promoters by Eσ70 and EσS, and indirect transcriptional activation by CsrA. Mol. Microbiol. *81*, 689–704.

Zimaro, T., Thomas, L., Marondedze, C., Sgro, G.G., Garofalo, C.G., Ficarra, F.A., Gehring, C., Ottado, J., and Gottig, N. (2014). The type III protein secretion system contributes to Xanthomonas citri subsp. citri biofilm formation. BMC Microbiol.

Figures

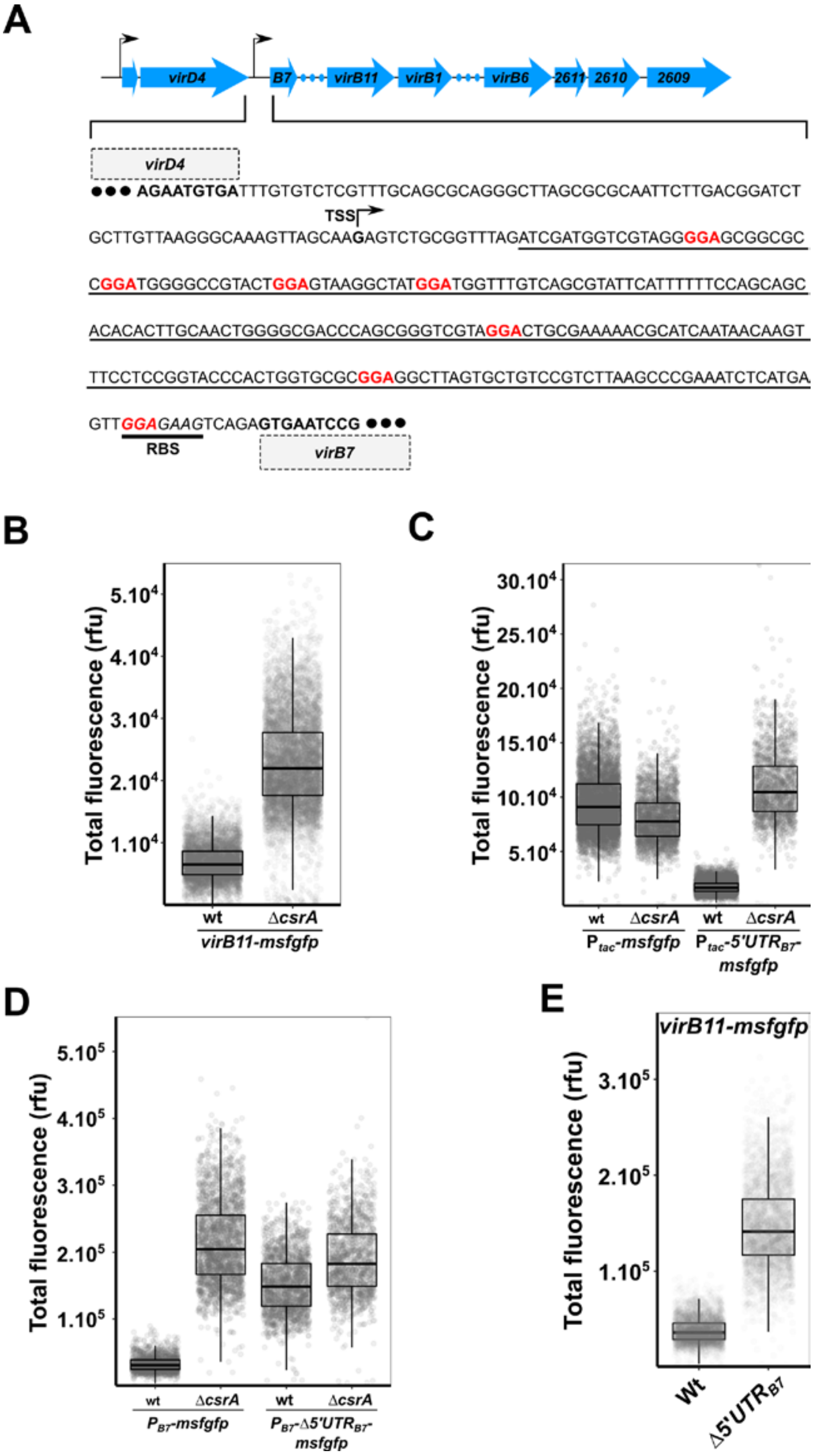

Figure 1. CsrA regulates the *virB* operon. A) Schematic of the *X. citri virD* and *virB* operons with black arrows indicating positions of the transcription start sites, blue arrows indicating

the open reading frames and blue dots representing genes not shown. The intergenic sequence between *virD4* and *virB7* genes is shown and the *virB7* transcription start site (TSS) is indicated. The 5´-GGA-3´ motifs are indicated in red and the predicted ribosome binding site (RBS) is underlined with a thick black line. The sequence that is deleted in the *Δ5´UTR*$_{B7}$ strains is underlined with a thin black line. B) Single-cell msfGFP fluorescence levels of the *X. citri virB11-msfgfp* transcriptional reporter in *X. citri* wild-type and *ΔcsrA* strains. Production of msfGFP increased on average 3.3-fold in the *ΔcsrA* strain (N_{wt} = 4887 cells and $N_{\Delta csrA}$ = 6263 cells, from a single representative culture each). C) Single-cell msfGFP fluorescence levels of the *amy::P*$_{tac}$*-msfgfp* and *amy::P*$_{tac}$*-5´UTR*$_{B7}$*-msfgfp* reporters in *X. citri* wild-type and *ΔcsrA* strains. A CsrA-dependent reduction in msfGFP production is only observed in the strain containing the *5´UTR*$_{B7}$ (average 5.8-fold reduction, N_{wt} = 6862 cells and $N_{\Delta csrA}$ = 1671 cells, from 2 separate cultures each). The control lacking the *5´UTR*$_{B7}$ in between the P_{tac} promotor and *msfgfp* (*X.citri amy::P*$_{tac}$*-msfgfp*) showed no CsrA-dependent repression of msfGFP production (average 16% increase, N_{wt} = 5534 cells and $N_{\Delta csrA}$ = 2495 cells, from 2 separate cultures each). D) Single-cell msfGFP fluorescence levels of the *msfgfp* reporter that substituted the entire structural *virB* operon (with the *msfgfp* start codon placed at the exact position of the *virB7* start codon) in *X. citri* wild-type and *ΔcsrA* strains with an intact *virB* promoter (*P*$_{B7}$) or a *virB* promoter in which the *5´UTR*$_{B7}$ was deleted (*P*$_{B7}$*-Δ5´UTR*$_{B7}$). *X. citri ΔvirB::P*$_{B7}$*-Δ5´UTR*$_{B7}$*-msfgfp* shows higher msfGFP production than the *X. citri ΔvirB::P*$_{B7}$*-msfgfp* strain containing the wild-type 5´UTR$_{B7}$ (average 6.5-fold increase, N_{wt} = 1495 cells and $N_{\Delta csrA}$ = 2156 cells, from 2 separate cultures each). Absence of the *5´UTR*$_{B7}$ almost completely abolishes CsrA dependent down-regulation of expression levels (average 1.2-fold increase, N_{wt} = 1748 cells and $N_{\Delta csrA}$ = 1304 cells, from 2 separate cultures each). Note that removal of the *5´UTR*$_{B7}$ in the wild-type backgrounds led to a 4.6-fold increase. E) Single-cell msfGFP fluorescence levels from the *X. citri virB11-msfgfp* reporter strain with and without the genomic deletion of the *5´UTR*$_{B7}$ (underlined region in part A; *X. citri Δ5´UTR*$_{B7}$ *virB11-msfgfp*). Removing the *5´UTR*$_{B7}$ leads to a 3.9-fold increase in msfGFP production from the *virB* operon (N_{wt} = 3397 cells and $N_{\Delta 5'UTR}$ = 3099 cells, from 4 separate cultures). Tukey box-and-whisker plots in parts B-E: black central line (median), box (first and third quartiles) and whiskers (data within 1.5 interquartile range).

Figure 2

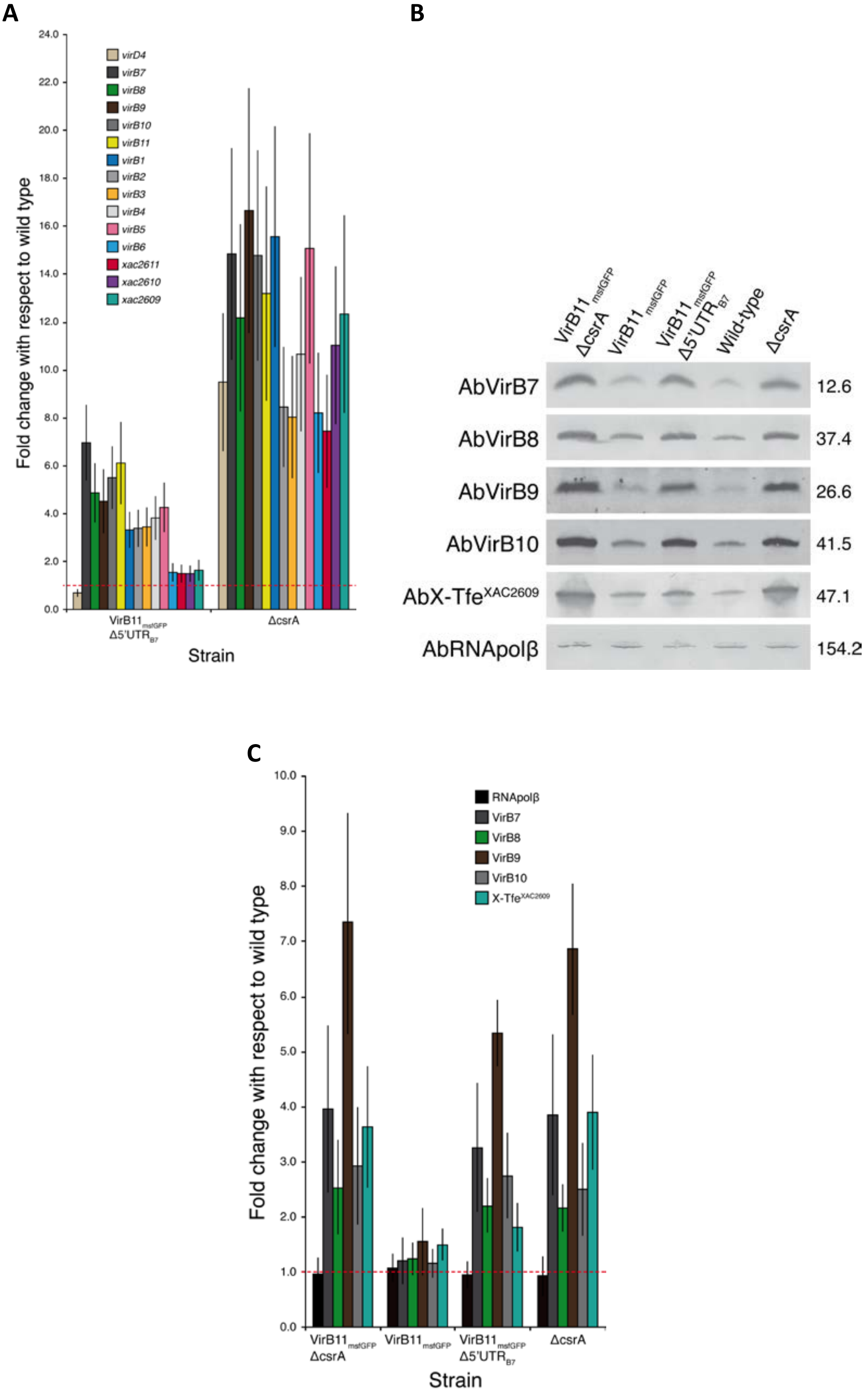

Figure 2. Quantitative analysis of X-T4SS gene expression and protein levels in *X. citri.* A) X-T4SS gene expression analysis in different genomic backgrounds. Quantitative real-time

polymerase chain reaction (RT-qPCR) of representative genes within the *X. citri* X-T4SS *virD* and *virB* operons. Vertical bars represent the mean value from four RT-qPCR runs (two biological and two technical replicates for each gene). Vertical lines represent the standard deviations. The 16S gene was used as an internal reference housekeeper gene. The *X. citri* wild-type strain (fold change = 1.0 represented by the dashed horizontal red line) was used as the reference sample. B) Western blot band intensity as a measurement of protein accumulation in *X. citri* total cell lysates. Assays were performed using polyclonal antibodies (Ab) against specific *X. citri* X-T4SS structural components or effector (X-Tfe^{XAC2609}) in different *X. citri* strains (see Materials and Methods). To adjust for small differences in sample loading and transfer, monoclonal antibodies against the β subunit of RNA polymerase (AbRNApolβ) were used. Experiments were repeated five times with similar results. Full western blot membranes are presented in Supplementary Figure 4. Theoretical molecular weight (in kDa) for each mature protein is shown at the right. C Quantitative analysis of protein accumulation. Band intensities from membranes, as in B, were normalized to the values of *X. citri* wild-type strain (fold change = 1.0 represented by the dashed horizontal red line) for each assayed protein (see Materials and Methods). Bars represent the mean value from at least four experiments (minimum two biological replicates) and vertical error bars represent the standard deviation.

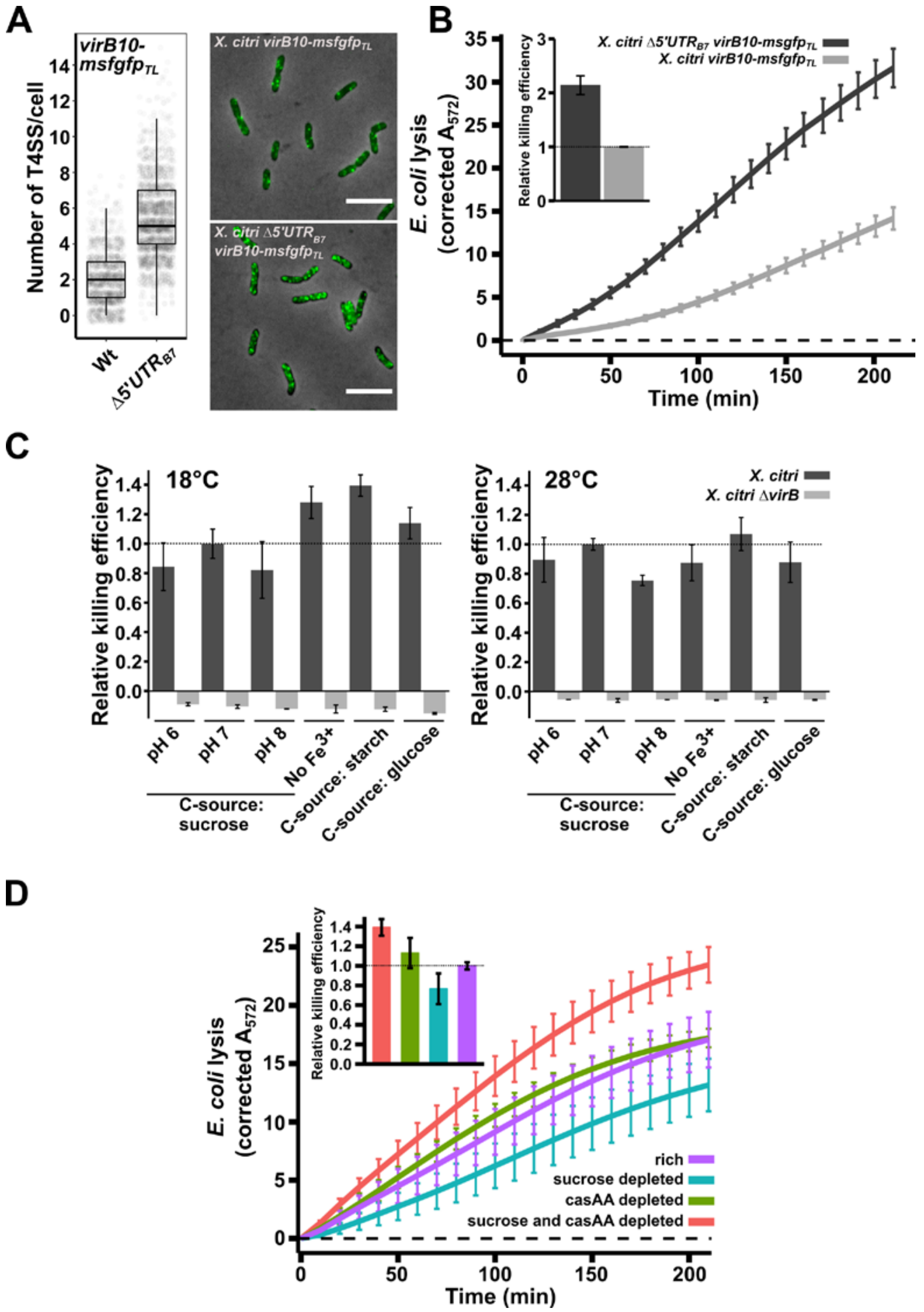

Figure 3. X-T4SS production and interbacterial killing is constitutive under incomplete repression by CsrA. A) Quantification of single fluorescent foci of X-T4SSs containing the VirB10-msfGFP chimera. The genomic deletion of the *5´UTR*$_{B7}$ in the *X. citri virB10-msfgfp*$_{TL}$ strain increased the number of X-T4SSs per cell 2.6-fold (N_{wt} = 1361 cells and $N_{\Delta 5'UTR}$ = 2062

cells, from 3 separate cultures). Due to occasional overlap of the greater number of fluorescent foci in the *Δ5´UTR*$_{B7}$ strain, the number of T4SS per cell may be underestimated in this strain. B) Quantitative CPRG cleavage-based killing assay in the presence or absence of CsrA repression on the *virB* operon using the same *X. citri virB10-msfgfp*$_{TL}$ and *X. citri Δ5´UTR*$_{B7}$ *virB10-msfgfp*$_{TL}$ cultures analysed in part A. Corrected A_{572} values are given in arbitrary units (see Materials and Methods). The inset shows the normalized slope value of the linear part of the depicted curves relative to the wild-type strains (N = 4 separate cultures with 2 technical repeats each). Genomic deletion of the *5´UTR*$_{B7}$ leads to a 2.14-fold increase in the rate of lysis of *E. coli* cells. C) Quantitative CPRG cleavage-based killing assays of wild type *X. citri* cells grown in different conditions (dark grey bars). Killing efficiencies were evaluated in defined media containing different carbohydrate sources (0.2 % sucrose, 50 µg/ml starch or 0.2% glucose), pH 6, 7 and 8, lack of Fe^{3+} and at different temperatures (18°C and 28°C). Values represent the slope of the linear part of the OD_{572} curves as described in part B and normalized relative to the condition at pH 7 (which is the condition used throughout the manuscript). As a control, killing efficiency of a T4SS-deficient mutant (*X. citri ΔvirB::P*$_{B7}$*-msfgfp*; light grey bar) was assessed after growth under the same conditions (N = 4 separate cultures with two technical repeats). D) Quantitative CPRG cleavage-based killing assays from *X. citri* cells grown at different nutrient levels. Killing efficiencies of wild type *X. citri* strains are maintained during growth in defined media containing either 0.2% or 0.01% sucrose and/or casamino acids (casAA). Inset shows the slope value of the linear part of the depicted curves normalized with respect to the killing curve from the *X. citri* cultures grown in rich media (relative killing efficiency = 1.0, horizontal line). Cultures depleted in both casamino acid and sucrose concentrations show a 39% increase whereas sucrose-depleted cultures have a 23% reduction in efficiencies compared to the reference (N = 5 separate cultures with 4 technical repeats). Error bars in panels B, C and d indicate the standard deviation. Horizontal dashed lines in panels B and D represent the zero-line obtained for the background signal unchallenged (non-lysed) *E. coli* cultures grown in parallel during each experiment.

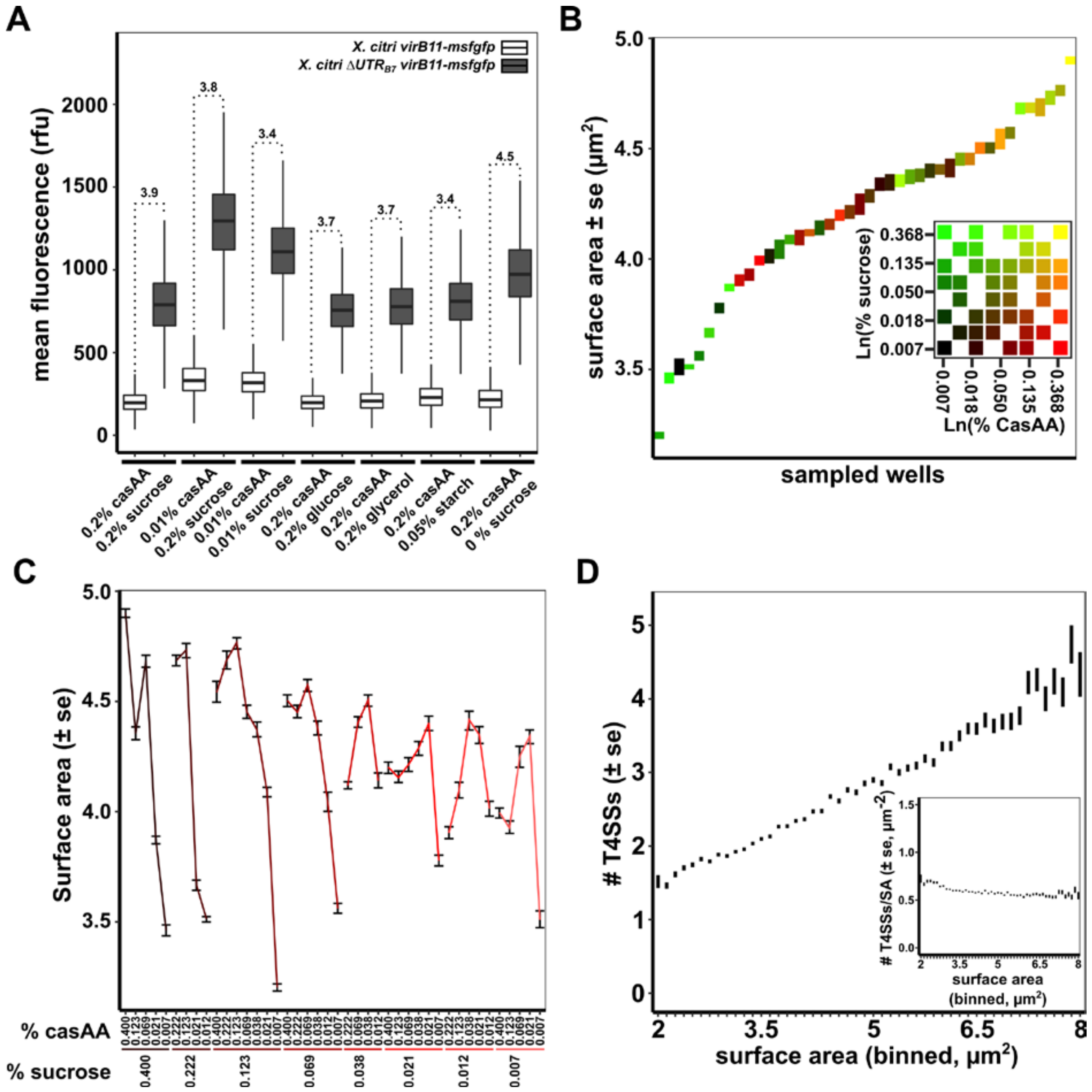

Figure 4. X-T4SS production and density in the cell envelope under different nutrient conditions. A) msfGFP fluorescence levels from *X. citri virB11-msfgfp* and *X. citri Δ5´UTR$_{B7}$ virB11-msfgfp* grown in AB defined media containing either sucrose, glucose, glycerol, starch or no carbohydrate source (0% sucrose) in combination with 0.2% casamino acids (casAA) or in AB defined media containing 0.01% casamino acids with 0.2% or 0.01% sucrose. Mean cytoplasmic msfGFP production from the *X. citri virB11-msfgfp* transcriptional fusion strain in the presence (white boxplots) or absence (grey boxplots) of the *5´UTR$_{B7}$* are represented as Tukey box-and-whisker plots for each media composition with black central line (median), box (first and third quartiles) and whiskers (data within 1.5

interquartile range). The *Δ5´UTR*$_{B7}$ *virB11-msfgfp vs virB11-msfgfp* ratio of the mean fluorescence levels is given above the Tukey plots for each growth condition. On average 6018 cells were sampled per condition for each strain, with a minimum of 2162 and a maximum of 16351 cells from two independent cultures. Note that in this graph, the mean fluorescence (sum of each cell's pixel intensities divided by the number of pixels) is used to take into account differences in cell-sizes between conditions. B) Surface area of *X. citri* cells sampled from separate cultures, each culture differing slightly in sucrose and casamino acid concentrations. Cultures are ordered according to the average cell surface area observed for each growth condition. The inset shows the 8x8 matrix range of sucrose and casamino acid concentrations used as described in the text. In both the graph and the inset, each growth condition is colored differently. C) Surface area of *X. citri* cells as a function of sucrose and casamino acid concentrations. The data is the same as in part B but organised with respect to sucrose and casamino acid concentrations and illustrates the link between *X. citri* cell sizes and the culture media composition. D) Average number of T4SSs per cell versus cell surface area from all the cells grown in the different conditions presented in panels B and C. Detection of VirB10-msfGFP foci reveals that the increase in average number of T4SSs correlates with increasing surface area (Pearson correlation $r = 0.34$, as calculated for all data points). *Inset:* Density of T4SSs in the cell envelope (number of T4SS per surface area, T4SS/SA) versus the surface area. A subtle increase with smaller (and thus more nutrient deprived) cells is observed. Vertical bars in parts B, C and D represent the standard error around the mean (± standard error). In total 69412 cells were registered for the data in parts B, C and D.

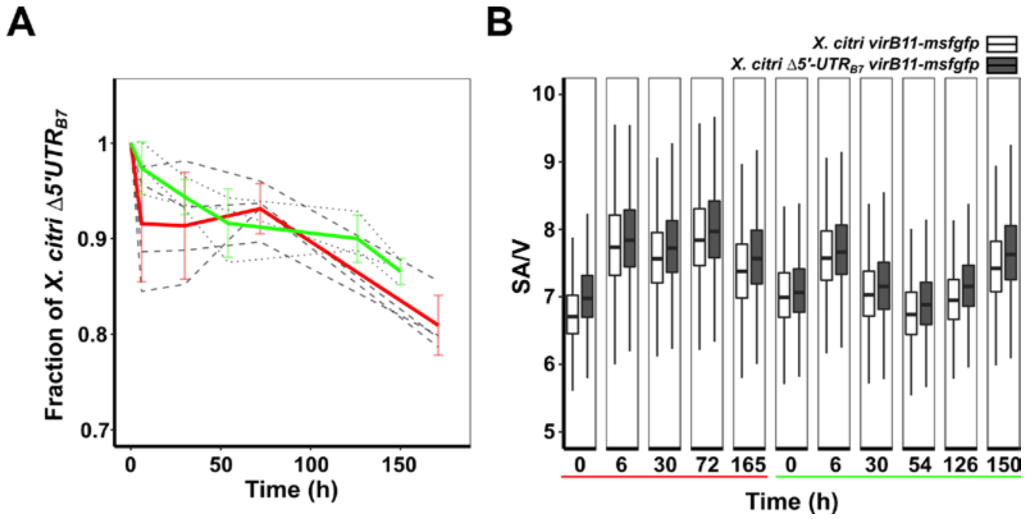

Figure 5. Overproduction of X-T4SSs leads to a detectable physiological cost for *X. citri*. A) Co-culture experiment between *X. citri virB11-msfGFP* and X. *citri Δ5´UTR$_{B7}$ virB11-msfGFP* in liquid medium. A decrease in the fraction of *X. citri Δ5´UTR$_{B7}$ virB11-msfGFP* cells relative to the wild-type (*X. citri virB11-msfGFP*) cells is observed and becomes pronounced after approximately 1-week co-incubation. Results from two distinct experiments with three (dotted lines and green mean values) or four (dashed lines and red mean values) separate cultures are shown. In all seven cultures, we observed a significant reduction in the fraction of *X. citri Δ5´UTR$_{B7}$ virB11-msfgfp* cells relative to the wild-type *X. citri virB11-msfgfp* cells at the end-points: 0.87 ± 0.01 (green) and 0.81 ± 0.03 (red). Cells were imaged by fluorescence microscopy and separated by the difference in msfGFP production levels as seen in Figure 1E. B) Comparison of surface area to volume ratios of *X. citri virB11-msfGFP* and *Δ5´UTR$_{B7}$ virB11-msfGFP* for cells collected at different times for all seven cultures shown in part A. A subtle but consistently smaller average cell size (larger SA/V) in the *Δ5´UTR$_{B7}$* background is observed, indicative of a subtle change in physiological state of the cells that can be attributed to the increased production of X-T4SSs. At each time point, an average of 3871 cells were analysed for each culture (minimum: 1061 cells, maximum: 9582 cells).

Supplementary Figures and Tables

Bactericidal Type IV Secretion System Homeostasis in Xanthomonas citri

William Cenens1, Maxuel O. Andrade2, Edgar Llontop1, Cristina E. Alvarez-Martinez3, Germán G. Sgro1 and Chuck S. Farah1

A

Transcription start site of *virD4*

GGTCAGCGCTGCCTACCGCAGCCCAGTCCGCCGATACC**GCTTTC**ACCGTCACACGTCTTGCT
T**TAGCAT**CGCCACC**GC**GCTGACGGAAAGGAAGGCTGGCG**ATG**GACGAATATCAGCACACTGT
GCTGACCAGGGGGGGTACCGTGTTGTGGCGATTACGCGTGACGAGGTGTACGCACCCGACG
CCGTCGTGGCATACGCCGTTGTGACCGACGCGGGTACGCGAATCACTCCGGATTTGTCCCTC
GACCAGGCCAAGGTCTGGATCGATTCGCTGGTGGAGAGCGAGAGTGGCGGGCGCAAGTCCGG
CCTTGTCGACCATAAGCCGGTCGTGCGTCGCTAGCCCGCCATCGTGATGCAAGCCTAAGGAG
AAGGGCGC**ATG**TCCAAACCCAAAATCATCGCAATTGCCACGCTGGTGG

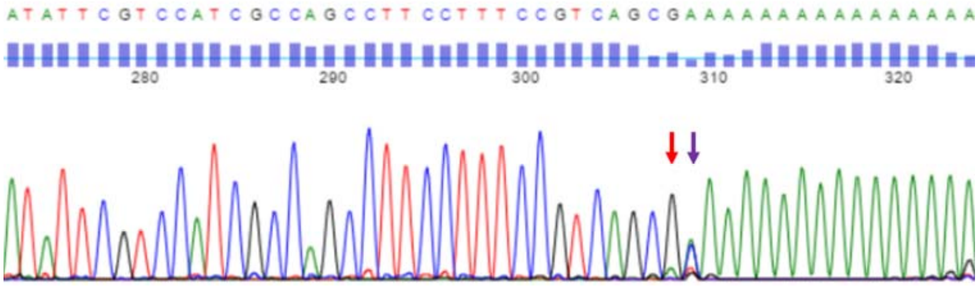

B

Transcription start site of *virB7*

TGTCTCGTTTGCAGCGCAGGGCTTAGCGCGCAATT**CTTGAC**GGATCTGCTTGTTAAGGGC**AAAGTT**
AGCAA**G**AGTCTGCGGTTTAGATCGATGGTCGTAGGGGAGCGGCGCCGGATGGGGCCGTACTGGAGT
AAGGCTATGGATGGTTTGTCAGCGTATTCATTTTTTCCAGCAGCACACACTTGCAACTGGGGCGAC
CCAGCGGGTCGTAGGACTGCGAAAAACGCATCAATAACAAGTTTCCTCCGGTACCCACTGGTGCGC
GGAGGCTTAGTGCTGTCCGTCTTAAGCCCGAAATCTCATGAGTTGGAGAAGTCAGA**GTG**AATCCGA
TGTATGTGTCCAAGCTGTCGTTGGTGCTAGTGGCTG

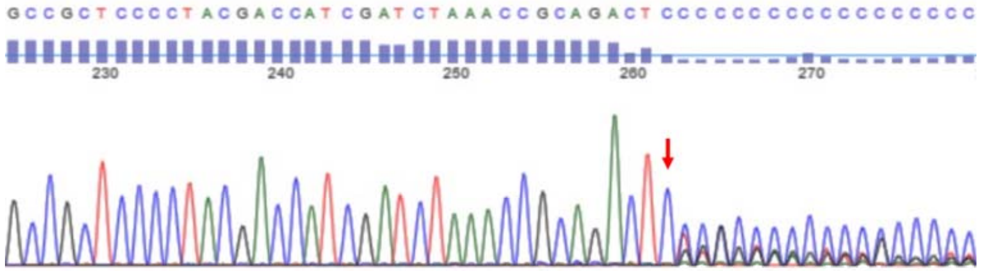

Supplementary Figure 1. 5´RACE assay identifying the transcription start sites for *virD4* and *virB7*. A) Bottom panel shows the two adjacent possible transcription start sites (TSS) of *virD4* (red and purple arrow) due to sequencing ambiguity. The possible start sites are indicated with a purple G and a red C in the nucleotide sequence (top panel). The *virD4* sequence starts at the end of the displayed nucleotide sequence. In between the TSS and *virD4* a putative open reading frame (ORF) coding for a protein of unknown function can be found. B) Similar analysis for the *virB7* with TSS depicted with red arrow (bottom panel) and red G in the nucleotide sequence (top panel). In parts A and B, translation start sites are depicted in red and open reading frames in blue. Putative RNA polymerase binding sites are shown in bold and putative ribosome binding sites are underlined in regular font.

Supplementary Figure 2

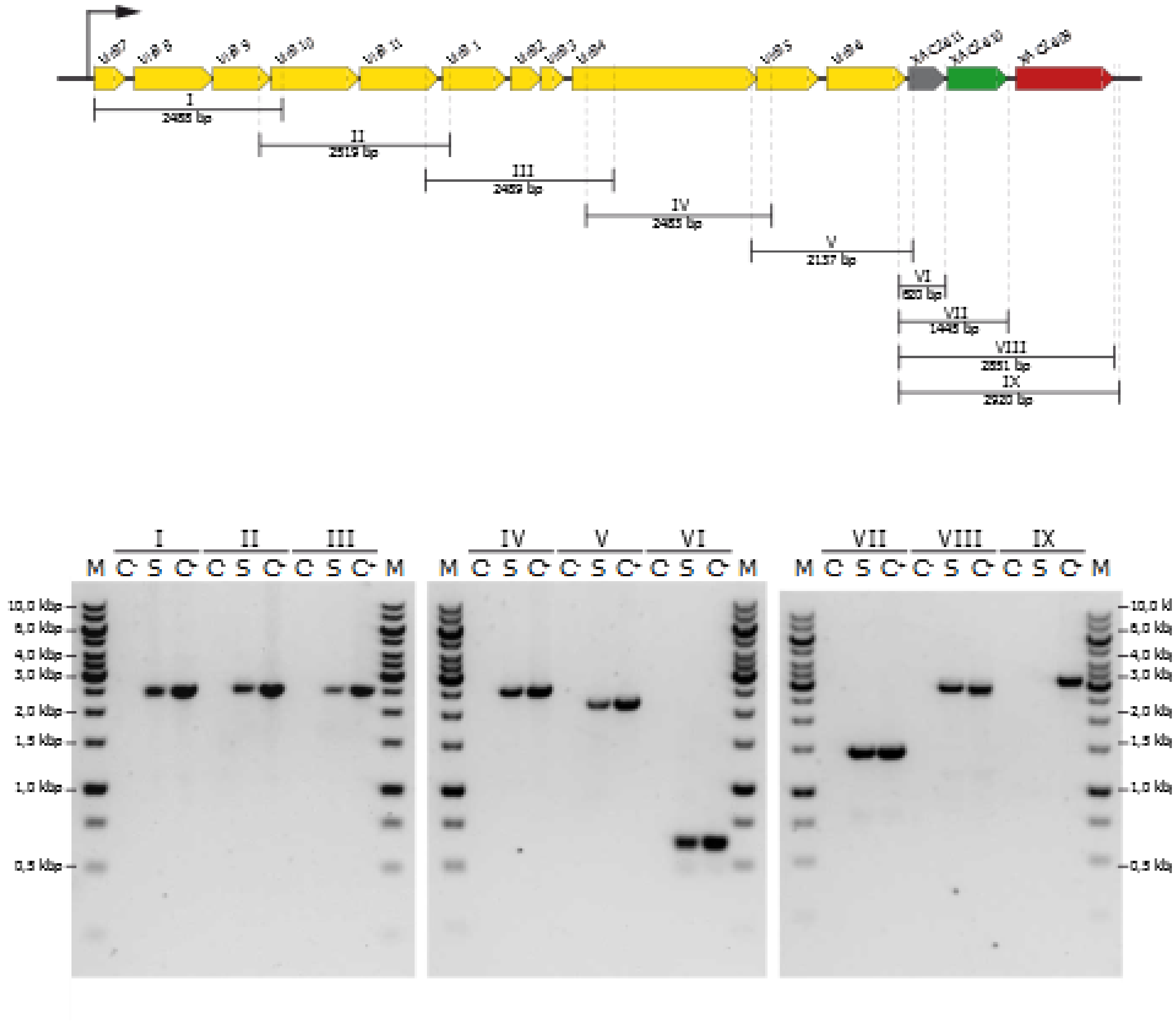

Supplementary Figure 2. *X. citri virB* operon transcriptional analysis. The *upper panel* shows a diagram of *X. citri virB* operon, from *virB7* to *xac2609*. A black arrow indicates the promoter proposed for gene transcription, located upstream of *virB7*. Genes coloured in yellow correspond to structural T4SS components, in grey to hypothetical proteins, in green to X-Tfis, and in red to X-Tfes. Nine sets of primers (designated I to IX) amplifying overlapping regions were designed in order to determine the length of the polycistronic mRNA transcript (see Materials and Methods). The *lower pane*l shows pictures of SYBR Safe stained 1% agarose gels in which the PCR products (depicted in the *upper panel*) were applied. C-: negative control, non-reverse transcribed *X. citri* RNA template; S: sample, reverse transcribed *X. citri* RNA template; C+: *X. citri* genomic DNA template.

Supplementary Figure 3

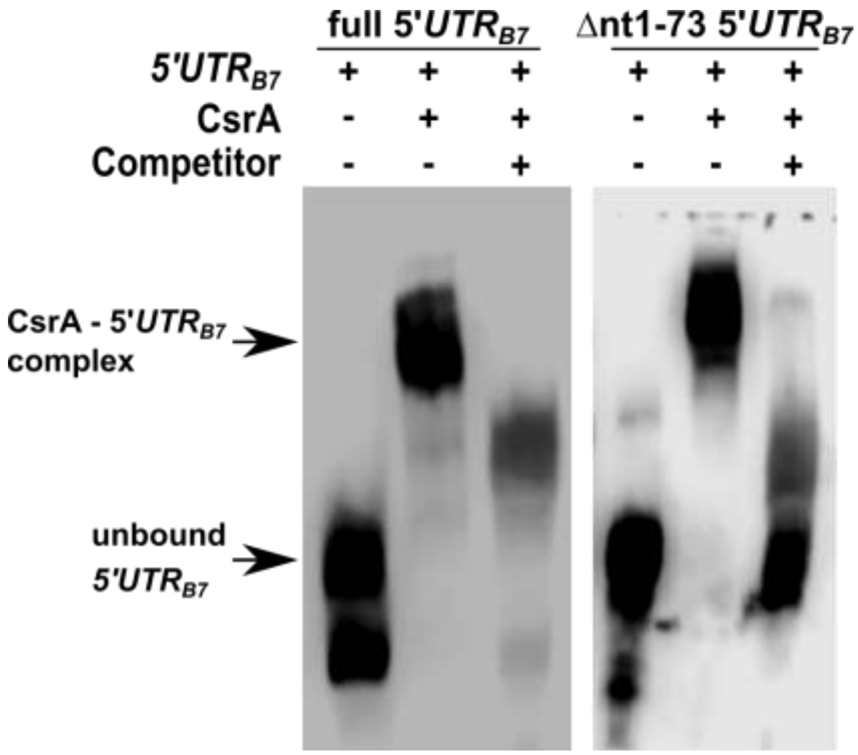

Supplementary Figure 3. Electrophoresis mobility shift assay (EMSA). EMSA shows direct *in vitro* binding of CsrA with the complete *5´UTR*$_{B7}$ and a shortened Δnt1-73 *5´UTR*$_{B7}$ fragment (lacking the first 73 nucleotides, up to and including the fourth GGA motif). See Figure 1 from the main text for the *5´UTR*$_{B7}$ sequence. Binding reactions were performed using 70 nM of purified CsrA protein and 6.25 nM Biotin-labelled RNA. Addition of 312.5 nM unlabelled *5´UTR*$_{B7}$ RNA competes with labelled RNA binding. See Materials and Methods for details.

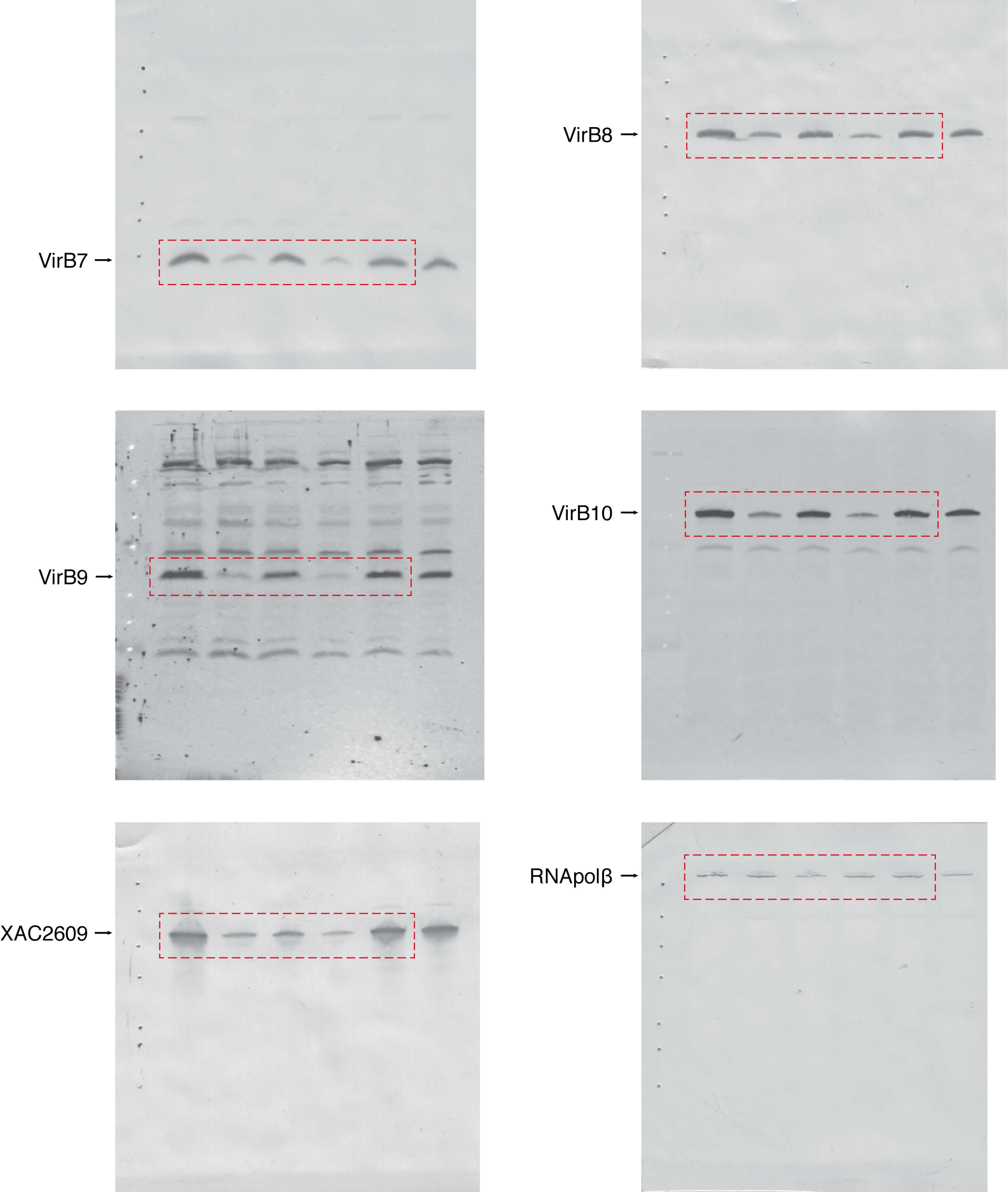

VirB7
VirB8
VirB9
VirB10
XAC2609
RNApolβ

Supplementary Figure 4. Whole nitrocellulose membranes employed for the preparation of Figure 2B. Polyclonal specific antibodies (Ab) against VirB7, VirB8, VirB9, VirB10, and XAC2609 were used. In the case of the β subunit of RNA polimerase (RNApolβ), monoclonal antibodies were used (see Materials and Methods for details). Experiments were repeated five times for VirB7, VirB8, VirB9, VirB10, and XAC2609 and four times for RNApolβ showing similar results. Expected molecular weights for mature proteins are: VirB7 12.62 kDa, VirB8 37.38 kDa, VirB9 26.56 kDa, VirB10 41.53 kDa, XAC2609 47.10 kDa, and RNApolβ 154.20 kDa. Note that VirB10 is a proline-rich protein, therefore it presents an apparent higher molecular weight.

Supplementary Movie Legends

Supplementary Movie 1. Time-lapse movie showing contact dependent lysis at the single-cell level during growth in media depleted for casamino acids (AB medium+ 0.2% sucrose + 0.01% casamino acids). Movie starts after 25 hours of growth on the pad due to significantly reduced growth speeds. White arrows indicate regions were *E. coli* cells come into contact with the smaller sized *X. citri* cells. Scalebar: 5µm. Time stamp in bottom left of the movie. See Materials and Methods for details of growth conditions.

Supplementary Movie 2. Time-lapse movie showing contact dependent lysis at the single-cell level during growth in rich media (AB medium + 0.2% sucrose + 0.2 % casamino acids). Movie was initiated in parallel with Supplementary Movie 1, starting at time point zero. White arrows indicate regions were *E. coli* cells come into contact with smaller sized *X. citri* cells. Scalebar: 5µm. Time stamp in bottom left of the movie. See Materials and Methods for details of growth conditions.

Table S1. Primers, strains and plasmids used in this study

Primer Cloning purpose	Primer name	Sequence 5' - 3'	Description
**Construct in pNPTS138 for transcriptional fusion *virB11-msfgfp***			
	CW003	TCAG **GAATTC** *GGAGTGAAACGATG* AGTAAAGGTGAAGAACTGTTCAC	Introduce ribosome binding site and start codon to *msgfp* in pDHL1029. Forward primer.
	CW004	TCAG **CCCGGG** TTATTTGTAGAGTTC	Introduce ribosome binding site and start codon to *msgfp* in pDHL1029. Reverse primer.
	CW005	TCAG **AAGCTT** CGCCTGACGTGACCG	Reverse primer 1. For upstream target. Ligates to pNPTS138.
	CW006	TCAG **GAATTC** CTAGCCGCCGCGCAG	Forward primer 1. For upstream target. Ligates to *msgfp* .
	CW007	TCAG **CCCGGG** GGCATCGAGTTCGTCGCTG	Reverse primer 2. For downstream target. Ligates to *msgfp* .
	CW008	TCAG **GGATCC** GTCCAGCAGCCATATCCTTG	Forward primer 2. For downstream target. Ligates to pNPTS138.
	CW048	TACAGT **GAATTC** GGAGTGAAAC	Forward primer for amplifying *msgfp* with ribosome binding site from pDHL-*RBS-msfgfp* .
	CW004	TCAG **CCCGGG** TTATTTGTAGAGTTC	Reverse primer for *amplifying msgfp* with ribosome binding site from pDHL-*RBS-msfgfp.*
	CW061	CCATGACGATCGATGACTC	Confirmation of genomic *virB11-msfgfp* construct. Forward primer.
	CW062	GCAATGCCTTTACTGCCTG	Confirmation of genomic *virB11-msfgfp* construct. Reverse primer.
**Construct in pNPTS138 for *csrA* deletion strain**			
	CW040	TATCAG **GAATTC** CCTGATCGACTGCGCTTTG	Forward primer 1. For downstream target. Ligates to pNPTS138.
	CW041	TTCATG **CTCGAG** TACCCGGCGAGTGAGGATC	Reverse primer 1. For downstream target. Ligates to upstream target.
	CW042	AATGTC **CTCGAG** TCGAATTGATCGTTGTCAAGG	Forward primer 2. For upstream target. Ligates to dosnwstream target.
	CW043	TTCATG **AAGCTT** GAACGCCGAACATCGATAGG	Reverse primer 2. For upstream target. Ligates to pNPTS138.
	CW072	GTACTGGGAACGCATGTGC	Confirmation of genomic deletion of *csrA* . Forward primer.
	CW073	GTCCTGAGTGCCATTCATGG	Confirmation of genomic deletion of *csrA* . Reverse primer.
**Construction of *UTR* $_{B7}$ construct in between P$_{TAC}$ and msfGFP in pPM7G**			
	CW244	**CTTCCTTTCCGTCAGCG** CCACACATTATACGAGCCGATG	Reverse outward primer for pPM7G. Includes the PTAC promotor. Homology for unstructered small RNA with ribosome binding site. Homology with primer CW246
	CW245	CTGGCGTTACCCAACTTAATCG	Forward outward primer fo pPM7G. Downstream of pPM7G cloned GFP construct.
	CW246	**CGCTGACGGAAAGGAAGGCTGGCGATG** AGTAAAGGTGAAGAACTGTTC	Amplifies msfGFP with unstructured small RNA. Homology with primer CW244. Includes start for GFP. Forward primer.
	CW247	**CGATTAAGTTGGGTAACGCCAG** *TTA* TTTGTAGAGTTCATCCATGC	Amplifies msfGFP with homology to primer CW245. Reverse primer
	CW248	CCACACATTATACGAGCCGATG	Reverse outward primer for pPM7G. Includes the PTAC promotor. Use with CW245.
	CW249	**ATTAATCATCGGCTCGTATAATGTGTGG** GAGTCTGCGGTTTAGATCGATG	Amplifies UTR$_{B7}$ with homology to primer CW248. Forward primer.
	CW250	**ACCGGTGAACAGTTCTTCACCTTTACT** CACTCTGACTTCTCCAACTCATG	Amplifies UTR$_{B7}$ with homology to primer CW30. Includes start site of *virB7* serving as start for GFP and *virB7* ribosome binding site. Reverse primer
	CW030	AGTAAAGGTGAAGAACTGTTC	Amplifies msfGFP with no start site. Forward Primer. Reverse primer is CW247.

	CW063	GCACCATCATCACCAATCAC	Control primer to confirm *virB* integrity. Forward primer.
	CW064	GCATTTGATCAGGAAATGCTG	Control primer to confirm *virB* integrity. Reverse primer
**Construct in pNPTS138 for deleting all *virB* genes and *xac2609* , *xac2610* and *xac2611,* replacing operon with msfGFP under control of P_{virB} with or without UTR_{B7}**			
	CW216	**AGGATATCTGGATCCACGAATTCGCTAG** CTAGGAGCAGTGTCGGTATCG	Amplifies downstream target. pNPTS138 homology. Reverse 1 primer. Use with CW217.
	CW217	**TCACGCACGGCATGGATGAACTCTACAAATAA** CGTTGCACTTCAGCGAGC	Amplifies downstream target. *msfGFP* homology. Forward 1 primer.
	CW239	ACCGGTGAACAGTTCTTCACCTTTACT **CAC**TCTGACTTCTCCAACTCATG	Amplifies upstream target. *msfGFP* homology. Reverse 2 primer. Places *msfGFP* start at *virB7* start.
	CW240	TCACTTAAGGCCTTGACTAGAGGGTCGA GACCTTCGCCTCGATCATG	Amplifies upstream target. pNPTS138 homology. Forward 2 primer.
	CW271	AGTTCTTCACCTTTACT CACTCTGACTTCTCCAA CTAAACCGCAGACTCTTGC	Amplifies upstream target. *msfGFP* homology. Reverse 2 primer. Also deletes UTR_{B7} but maintains *virB7* ribosome binding site and *virB7* start codon for *msfGFP* . Use with primer CW240.
	CW031	TTTGTAGAGTTCATCCATGC	Amplifies *msfGFP* . Use with primer CW030. Assembles inbetween upstream and downstream targets.
	CW224	CAGAATCTGGCCCACACTG	Confirmation of genomic deletion of entire virB operon. Forward primer. Use CW063 as reverse primer.
**Construct in pNPTS138 for specifically deleting UTR_{B7} leaving rest of *virB* operon intact**			
	CW277	**TCACTTAAGGCCTTGACTAGAGGGTCGA** GAGACCGTGTTCCAGTCAC	Forward primer 1. Amplifies downstream target. Homology to pNPTS138.
	CW278	**GGGCAAAGTTAGCAAGAGTCTGCGGTTTAG** GTTGGAGAAGTCAGAGTGAATC	Reverse primer 1. Amplifies downstream target. Homology to primer CW279.
	CW279	**ATACATCGGATTCACTCTGACTTCTCCAAC** CTAAACCGCAGACTCTTGC	Forward primer 2. Amplifies upstream target. Homology to primer CW278.
	CW315	**AGGATATCTGGATCCACGAATTCGCTAG** GACCTTCGCCTCGATCATG	Reverse primer 2. Amplifies upstream target. Homology to pNPTS138.
			Use primers CW063 and CW064 for control of genomic deletion
**Internal primers for 5´RACE assay**			
	SP1-D4	GCAACCCAAGCGCGCTGAAATAGC	Nested primers for cDNA production of the *virD* transcript.
	SP2-D4	GAGCCGCAGCAACAGCAGGGTCAG	
	SP3-D4	CCACCAGCGTGGCAATTGCGATGA	Third primer for final PCR of the *virD* cDNA, identifying the TSS by sequencing.
	SP1-B7	ACGACCACCAAAGTCAGGAGCAGGCT	Nested primers for cDNA production of the *virB* transcript.
	SP2-B7	GCACGCGCCTACCAATGCTGCAGC	
	SP3-B7	CAGCCACTAGCACCAACGACAGCTTG	Third primer for final PCR of the *virB* cDNA, identifying the TSS by sequencing.
**Primers for cloning UTR_{B7} DNA fragments in pGEM-T**			
	F-B7	**TAATACGACTCACTATAGGG** AGTCTGCGGTTTAGATCGAT	Forward primer for PCR of the full length UTR_{B7}, containing the T7 promoter (bold font)
	F-B7-d73	**TAATACGACTCACTATAGGG** TGGTTTGTCAGCGTATTCATT	Forward primer for PCR of the shortened Δ1-73 UTR_{B7}, containing the T7 promoter (bold font)
	R-B7	ATCGGATTCACTCTGACTTCTCCAACTCATGA	Reverse primer for PCR of the different UTR_{B7} fragments

Overlapping analysis for determination of T4SS operon length

RT_Pair1_F	TGAATCCGATGTATGTGTCCAAG	Amplify 2488 bp for overlapping PCR Pair I.
RT_Pair1_R	GATCGGCTCATTGGCATCC	
RT_Pair2_F	GTCTTCGCTCGCGAGAAAG	Amplify 2519 bp for overlapping PCR Pair II.
RT_Pair2_R	TAGGGATTACGCGACGACTC	
RT_Pair3_F	GGACCAGCTTGCGTTGATG	Amplify 2489 bp for overlapping PCR Pair III.
RT_Pair3_R	AGAAAACCAGGCCGTTCTTTG	
RT_Pair4_F	AACACGTTCAACCGTCTTCTG	Amplify 2483 bp for overlapping PCR Pair IV.
RT_Pair4_R	TGGTCGTTCCATTGAGCTTG	
RT_Pair5_F	CAATGGTTGGCCGATTTCTATG	Amplify 2137 bp for overlapping PCR Pair V.
RT_Pair5_R	GGGCAGTCCAACTTACAAGAAG	
RT_Pair6789_F	TCCTCAAAGGGCACGTGG	Amplify 620 bp, 1445 bp, 2851 bp and 2920 bp for overlapping PCR Pairs VI, VII, VIII and IX, respectively. All these pairs share the same forward primer.
RT_Pair6_R	TCAATATTTCTTGAATATTGGATCAGAACAG	
RT_Pair7_R	CTAGTTGGACACCTCCTTCATTTC	
RT_Pair8_R	CCCGACTTACATGGACATTGAG	
RT_Pair9_R	TAGAGCCAAGGCGGACTTC	

RT-qPCR

RT_16S_F	tgcaactcgactccatgaag	Amplify 113 bp of 16S (used as house-keeping gene).
RT_16S_R	caacaaactcccatggtgtg	
RT_VirD4_F	tcatcaacctgctcttcagc	Amplify 108 bp of *virD4.*
RT_VirD4_R	aggtgaattcgtccatcagc	
RT_VirB7_F	atgtgtccaagctgtcgttg	Amplify 107 bp of *virB7.*
RT_VirB7_R	aaagtggttgacgtgcttcc	
RT_VirB8_F	acaagaactccacggtttcg	Amplify 117 bp of *virB8.*
RT_VirB8_R	gaaagcccagaggattttcc	
RT_VirB9_F	ttacagcaccggtttcactg	Amplify 121 bp of *virB9.*
RT_VirB9_R	ataagaatgcgtcgcggtac	
RT_VirB10_F	atattcgctgcatcctggag	Amplify 124 bp of *virB10.*
RT_VirB10_R	atcttggaccctttcggaag	
RT_VirB11_F	tcgaggtgatcaaacgtctg	Amplify 92 bp of *virB11.*
RT_VirB11_R	atcgatacccgtgatgaagc	
RT_VirB1_F	taatccctacgccattggtg	Amplify 117 bp of *virB1.*
RT_VirB1_R	cccagcgaaaagttgtaacc	
RT_VirB2_F	gaccaagctgttgaacatgg	Amplify 116 bp of *virB2.*
RT_VirB2_R	caatgagaattggcgagacc	
RT_VirB3_F	tttcaggctgctgggattac	Amplify 108 bp of *virB3.*
RT_VirB3_R	gggggattcttgcgatattc	
RT_VirB4_F	acaacaatccatggggtgac	Amplify 110 bp of *virB4.*
RT_VirB4_R	aatgagttctcggacggatg	
RT_VirB5_F	aggaagagcgcaagaagatc	Amplify 106 bp of *virB5.*
RT_VirB5_R	atctgctggcgatcgatatc	
RT_VirB6_F	ggctagagaaacgcaggatac	Amplify 106 bp of *virB6.*

RT_VirB6_R	aagcccacgattacctgaac	
RT_2611_F	tgtcggaagataaggcgaag	Amplify 109 bp of *xac2611.*
RT_2611_R	gaaaccaaagctgcacactg	
RT_2610_F	atggccaacggtctattctc	Amplify 115 bp of *xac2610.*
RT_2610_R	tcagcactgcctcgttattc	
RT_2609_F	gatcgtcaaaaccgctcttg	Amplify 118 bp of *xac2609.*
RT_2609_R	gcgtccgaaatcttcttcag	

**Plasmid**	**Description/Purpose**	**Reference**
pNPTS138	Contains a kanamycin Resistance gene, SacB and a pMB1 ori (suicide in *X. citri*). Standard plasmid used for creating specific genomic insertions or deletions by 2-step alelic exchanged.	Howard Steinman (Addgene plasmid # 41891)
pPM7G	Contains a kanamycin Resistance gene, a pMB1 ori (suicide in *X. citri*) and homology to the *amy* gene in *X. citri* . Constructs cloned in the MCS together with the plasmid are integrated into the *amy* gene in *X. citri* .	Martins et al., 2010
pPM7G-P_{tac}-*msfgfp*	contains *msfgfp* under control of the P_{tac} promoter.	This study
pPM7G-P_{tac}-5'UTR$_{B7}$-*msfgfp*	contains *msfgfp* under control of the P_{tac} promoter with the 5'UTR$_{B7}$ inbetween P_{tac} and *msfgfp*	This study
pGEM-T	Contains the T7 promoter upstream of the MCS for *in vitro* production of RNA from cloned fragments	Promega

pDHL1029	Contains template for *msfgfp* with a flexible linker for translational fusions	Ke et al., 2016
pDHL-*RBS-msfgfp*	Contains template for *msfgfp* with a ribosome binding site and start site for transcriptional fusions	This study

**Strain**	**Description/Purpose**	**Reference**
Xanthomonas citri pv. citri str. 306	Wild-type *X. citri* strain	da Silva et al., 2002.
Escherichia coli str. K-12 substr. M[illegible]	*Lac* positive *E. coli* strain for competition assays	Hayashi et al., 2006.
Escherichia coli str. Dh5α	Transformation strain for cloning purposes	Taxonomy ID: 668369
X. citri virB10::msfgfp $_{TL}$	The T4SS´s ViB10 protein linked to msfGFP. Constructed in the native allele.	Sgro et al., 2018
X. citri virB11-msfgfp	fusion of *msfgfp* in between *virB11* and	This study
X. citri virB11-msfgfp ΔcsrA	*X. citri virB11-msfgfp* with *csrA* deletion	This study
X. citri amy::P_{tac}*-msfgfp*	Insertion of pPM7G-P_{tac}-	This study
X. citri amy::P_{tac}*-5'UTR*$_{B7}$*-msfgfp*	Insertion pPM7G-P_{tac}*-5'UTR*$_{B7}$-	This study
X. citri amy::P_{tac}*-msfgfp ΔcsrA*	*X. citri amy::*P_{tac}*-msfgfp* with *csrA* deletion	This study
X. citri amy::P_{tac}*-5'UTR*$_{B7}$*-msfgfp* [illegible]	[illegible] *5'UTR*$_{B7}$*-msfgfp*	This study
X. citri ΔvirB::P_{B7}*-msfgfp*	Replacement of the entire *virB* operon (including effector pair *XAC2610* and *XAC2609*) with *msfgfp.* Placing the *msgfp* start cod	This study
X. citri ΔvirB::P_{B7}*-Δ5'UTR*$_{B7}$*-msfg*[illegible]	*X. citri ΔvirB::*P_{B7}*-msfgfp* with deletion of the 5'UTR$_{B7}$	This study
X. citri ΔvirB::P_{B7}*-msfgfp ΔcsrA*	[illegible] *ΔvirB::*P_{B7}-	This study
X. citri ΔvirB::P_{B7}*-Δ5'UTR*$_{B7}$*-msfg*[illegible]	*X. citri ΔvirB::*P_{B7}*-Δ5'UTR*$_{B7}$*-msfgfp* with *csrA* deletion	This study
X. citri Δ5'UTRB7 virB11-msfgfp	*X. citri virB11-msfgfp* with deletion of the 5'UTRB7	This study

Supplementary Movie 1

Click here to access/download
Supporting Information
Supplemental_Movie_S1.avi

Supplementary Movie 2

Click here to access/download

Supporting Information

Supplemental_Movie_S1.gif
