# Supplementary material for: Bactericidal type IV secretion system homeostasis in Xanthomonas citri
Source: PLoS Pathog. 2020 May 26;16(5):e1008561. doi: 10.1371/journal.ppat.1008561 (PMC7286519; doi:10.1371/journal.ppat.1008561)
Supplement: Attachment — Submitted filename: Response to Reviewers.docx [file ppat.1008561.s011.docx]

We are submitting the revised version of our manuscript " Bactericidal Type IV Secretion System Homeostasis in Xanthomonas citri" by Cenens, Andrade, Llontop, Alvarez, Sgro and Farah for publication in PLoS Pathogens. We thank reviewers for their constructive comments and suggestions in response to which we have conducted some additional experiments and analyses which can be found in Supplementary Figures 3 and 6. Our detailed responses **(in bold blue font)** to each of the reviewers' comments (in normal black font) can be found below.

We hope that the manuscript is now suitable for publication in PLoS Pathogens.

Sincerely,

Chuck Farah

Dept. de Bioquímica

Inst. de Química

Universidade de São Paulo

+++++++++++++++++++++++++++++++++++++++++++++++++++++++++++++++++

**Part I - Summary**
Please use this section to discuss strengths/weaknesses of study, novelty/significance, general execution and scholarship.

Reviewer #1: In this manuscript, Farah and his colleagues report on a characterization of the type IV secretion system (T4SS) in Xanthomonas that functions to inject toxic effectors into neighboring bacteria. The authors characterize the role of the transcriptional regulator CsrA in controlling production of the T4SS by: i) identifying potential CsrA binding sites in the upstream untranslated region (5’UTR) of the virB operon, ii) showing the CsrA binds the UTR of the virB operon, iii) demonstrating that a csrA deletion and mutation of the 5’ untranslated region (UTR) confer elevated virB expression, and iv) evaluating the effects of various growth conditions on T4SS production. Finally, they present evidence that CsrA regulates production of T4SS nanomachines within a certain range regardless of the environmental conditions tested, and that overproduction of these machines confers a fitness cost. Overall, the manuscript is well conceived and the findings support the conclusions drawn. The significance is mitigated by the fact that CsrA has been shown to regulate the production of many other translocation systems, the study does not provide new mechanistic insights into our knowledge of how CsrA controls virB transcription and translation, and there appear to be unexplored factors and regulatory networks other then CsrA that main T4SS homeostasis under different environmental/growth conditions. Nevertheless, the work clearly shows that CsrA functions as a transcriptional regulator that acts on the 5’ UTR of the virB regulon to inhibit overproduction of the T4SS.

**We thank the reviewer for the analysis and want to clarify the novelty this manuscript brings to the field. The novelty lies in the finding that CsrA repression provides *a basal and controlled level of expression* of that virB operon components (see, for example, lines 314-320, 366-367 and Figure 4A). As discussed in the third paragraph of the Discussion, experts in the field have suggested that CsrA could be an ideal candidate for such *homeostatic* responses. However, as far as we know, the outcome of CsrA regulation has not been studied from this point of view. Current regulatory effects of CsrA seem to have only been studied from the point of view of, for instance, a binary situation where CsrA aids in keeping expression levels to undetectable levels (OFF state) until certain conditions are met (ON state) or vice versa (see third paragraph of Discussion) or where conclusions have been drawn merely by regarding a CsrA deletion mutant. We also point out in the Discussion that strong on/off phenotypes are a possible outcome of CsrA regulation, but as we show here, CsrA regulation can also impose a homeostatic effect on specific operons such as the *virB* operon in *X. citri*. The experimental approaches at the single cell levels used in this study are capable of detecting such effects, whereas more traditional bulk-level experiments might have missed subtle basal expression and homeostatic effects. In conclusion, we hope that our work will encourage scientists to study CsrA regulatory effects in more detail at the single cell and single molecule level as this might reveal an unprecedented and widely conserved role in cellular house-keeping for CsrA.**

Reviewer #2: This manuscript reports a study of the regulatory function of CsrA (RsmA) on the type IV secretion machinery in Xanthomonas citri. All the experimental data shown in this manuscript clearly indicate that CsrA represses the expression of the T4SS and the accompanying cell-killing ability through the direct physical binding of the CsrA protein to the 5’UTR of virB7. Merits of this study include the employment of new techniques for better resolutions of the transcription data and improved visualization of the secretion machinery. Each experiment was designed in a very organized way and conducted rigorously. In spite of these merits, however, I could hardly find any new information that fills a knowledge gap in this area of science. The role of the T4SS in the bacterial competition of X. citri was previously discovered and the regulatory function of CsrA has been known in other bacterial systems. This study gives an impression that it was conducted to validate the previous microarray data through comprehensive experiments. If the authors want to publish this study in this journal, I strongly suggest that they clearly mention what point(s) of this study is the novel discovery that gives a scientific impact on the study of bacterial pathogens.

**We thank the reviewer this analysis and want to refer to the response we provided to reviewer #1 (above), where we argue and clarify the novelty of this study.**

Reviewer #3: The manuscript is well written and presents very interesting data relating to the control of the Vir genes by CsrA. The experiments appear well thought out as to the role of CsrA in regulation of the vir genes. In Figure 2B the Western blots indicate the protein concentrations for the various Vir proteins. I wonder how specific the antibodies are in order to make a quantitative assessment. Although you quantify surface area based on cell counts, are you stating in figure captions that the cell counts were per strain? The results section seems to contain discussion points. Perhaps you can combine the results and discussion given discussion points in results. I am not sure the surface/T4SS data are critical to this paper although I have no objection to it remaining. As for the co-cultivation, it would be more interesting to look at in planta comparisons rather than in vitro. Growing for ~50 hr to see a difference seems to be a stretch. Minor comments on the pdf file.

**We appreciate the reviewer's analysis.**

**In response to the individual questions raised here:**

- **Regarding the specificity of the antibodies: Supplementary Figure 5 provides images of the whole nitrocellulose membranes employed for the preparation of Figure 2B. Polyclonal antibodies (Ab) against VirB7, VirB8, VirB9, VirB10, and XAC2609 were used and all except for the antiVirB9 serum are quite specific.**
- **Cell surface area was quantified by taking the area (in pixels) of each single cell and calculating the surface area in µm^2^. In the experiment represented in figures 4B, 4C and 4D, 69412 cells were individually measured (Surface area, fluorescence and individual T4SSs). This large data set was presented in different ways: Fig 4B shows the results of the cells of each individual well of a 96 well plate; Fig4C shows the results for the cells grown in each specific condition; and the concluding figure 4D shows the results after binning all of the cells in discrete bins based on the calculated surface areas measured. In Fig 4B, 4C and 4D, one strain was used. In many other figures, the number of cells used was always given per strain or per condition or in the form of summary statistics. Thus, the reader is provided with a good idea of the amount of cells analyzed in each condition/strain.**
- **Regarding the separation of Results and Discussion, we believe that in some cases it is more adequate to add a phrase or two to explain a result or comment as to its relevance with respect to the recent literature instead of recapitulating the result in the Discussion in order to make the same point.**
- **When referring to the surface/T4SS data we assume that Reviewer #3 refers to the data as presented in figure 4. For this we would also like to refer to our reply to Reviewer #1 in the Summary section (above). Figure 4 and, most importantly, the data presented in Figure 4C, are crucial to the conclusions and novelty of this manuscript. These results show the remarkable observation that despite large differences in the growth media’s nutrient composition (which leads to large difference in cell sizes ­­and cell physiology), the total number of T4SS per unit surface area remains constant. And, as we point out in the presentation of the results shown in Figure 4 (subsection "Constant but incomplete CsrA-mediated repression of the virB operon during different growth conditions") this is the result of a constant repression of CsrA on the *virB* operon.**
- **Finally, we fully agree that a co-cultivation experiment in planta would be very interesting. However, the heterogeneity of such an experiment would make comparing the subtle differences as shown by the *in vitro* experiment of Fig 5, very difficult or even impossible to assess. We hope that the reviewer can appreciate that we are looking into physiological differences (cell size and fluorescence) that were made visible only by scrutinizing many thousands of single cells in well-controlled laboratory settings in which background fluorescence was reduced to a tolerable minimum. Nonetheless, we strongly suspect that in a real-world setting and over many generations, a *X. citri* strain with a hyper-active *virB* operon, despite having greater inter-bacterial killing efficiencies, would be outcompeted by a *X. citri* strain containing a CsrA controlled *virB* operon *in planta*. This point is now stated at the end of the second-last paragraph of the Discussion in the revised manuscript.**

**Part II – Major Issues: Key Experiments Required for Acceptance**
Please use this section to detail the key new experiments or modifications of existing experiments that should be absolutely required to validate study conclusions.

Generally, there should be no more than 3 such required experiments or major modifications for a "Major Revision" recommendation. If more than 3 experiments are necessary to validate the study conclusions, then you are encouraged to recommend "Reject".


Reviewer #1: 1. Studies reported in figure 5 should be carried out using strains tagged with different fluorescent markers, e.g., GFP, mRFP.

**The strength and ingenuity of this experiment is that by using equivalent markers in the two cell lines, the genetic differences are restricted *solely* to the presence or absence of the 5'UTR_B7_. The ability to distinguish the two populations is based on their differences in msfGFP expression levels by using single-cell techniques, which dispense the need for different markers and different strain constructions that could lead to unexpected effects on cell viability and fitness. Please note that these analyses are NOT based on T4SS focal production (as the reviewer seems to have misunderstood in the minor comment sections) but rather on the total amount of msfGFP (cytoplasmic msfGFP) that is produced due to the insertion (not fusion) of the msfgfp genes between the virB11 and virB1 ORFs in the virB operon (strain *virB11-msfGFP*). In order to clarify this, we have added the fluorescence intensity histograms of the co-cultures and of the separate individual cultures in Supplementary Figure 6. These histograms clearly show that the fluorescence intensity distributions of the two populations are found in two well-separated Gaussian curves at all time points.**

2. Further studies aimed at localizing the CsrA binding site within the UTR, testing for CsrA binding to DNA, and monitoring CsrA cellular levels in different growth conditions would help to define CsrA's mechanism of action.

**The full mechanistic insights of CsrA binding to 5’UTR loops containing GGA motifs are well studied and have shown specifically for *X. citri* CsrA in Andrade et al (2014). In order to address the reviewer's concern, we produced and placed a single copy *amy::P_tac_-5´UTR_B7_-gus* reporter in the *X. citri* wild-type strain (inserted in the amy gene) and determined the effect of introducing mutations in the reporter's *5'UTR_B7_* sequence to determine if one or more of the GGA motifs could contribute to this regulation. Supplementary Figure 3B shows that this reporter's expression is significantly up-regulated in the ΔcsrA background. Supplementary Figure 3C shows that deleting the first 72 nucleotides of the *5'UTR_B7_* that contains the first 4 GGA motifs, does not significantly affect GUS activity. The remaining 3 GGA motifs were mutated to GAA (strains 141-GAA, 184-GAA and 238-GAA). A ~1.7-fold increase in GUS activity was observed for the 141-GAA strain while no significant change in gene reporter activity was observed for the 194-GAA strain (Supplementary Figure 3C). A ~1.7-fold increase was also observed for a strain containing both 141-GAA and 194-GAA mutations. As could be expected, the 238-GGA mutation within the ribosome binding site, either on its own or in combination with 141-GAA, resulted in a drastic decrease in GUS activity (Supplementary Figure 3B), most likely due to compromised ribosome binding and translation initiation. Thus, we have evidence that part of CsrA's regulatory effect could be due to its binding to the GGA motif at position 141 of the transcript. Since the effect of this mutation (1.7-fold) was not as significant as that observed for the *∆csrA* strain (3-fold), we cannot rule out the possibility that CsrA is binding to other sites in the *5'UTR_B7_*, for example GGA motif within the RBS. This information is presented in the second-last paragraph of the first subsection of the Results section ("CsrA regulates the virB operon by binding to the 5´UTR of virB7")**

3. The physiology experiments should be repeated for the deltaUTR and deltaCsrA mutant strains.

**As pointed out in the second paragraph of the results section, the complex and pleiotropic effects of the *ΔcsrA* genotype on *X. citri* physiology would compromise any conclusion drawn from this kind of analysis. Therefore, the experiment suggested by the reviewer is not feasible nor would it lead to useful or interpretable results if it were carried out. It was exactly for this reason that we constructed a *Δ5'UTR* strain and showed with great care that this is the locus on which CsrA exerts its effect (see Figures 1C, 1D, 1E and 2A).**

Reviewer #2: N/A

Reviewer #3: (No Response)

**Part III – Minor Issues: Editorial and Data Presentation Modifications**
Please use this section for editorial suggestions as well as relatively minor modifications of existing data that would enhance clarity.


Reviewer #1: Comments:
1. Pg. 6. Can the authors exclude the possibility that CsrA binds the DNA corresponding to the 5’ UTR and not the RNA as a mechanism for controlling transcription or translation?

**CsrA is very well studied when it comes to the molecular nature of its interaction with RNA molecules (see references mentioned in the second-last paragraph of the Introduction). Furthermore, specifically in *X. citri*, it has also clearly been shown that CsrA binds 5’UTR RNA loops (Andrade et al, 2014). We do not know of any reports of DNA binding by CsrA or its orthologs.**

2. Pg. 6. Given that both the UTR’s of virD4 and virB encode proteins, it would seem important to knock these out with point mutations or small deletions to confirm that they don’t play a role in regulation.

### **The 5’UTR region of virB7 very likely does not encode a protein as pointed out in the first paragraph of the Results section. On its own, an open reading frame does not constitute the necessary requirements to encode a protein. RBS, distance to the promoter, sequence complexity and sequence conservation are all important factors. When taken together, we are relatively confident that no protein is encoded by the virB 5’UTR_B7_. The UTR of VirD4, however, does encode a protein that is currently under investigation in our laboratory and will be discussed in a future publication.**

3. Pg. 6. The deltaUTR mutation doesn’t phenocopy the deltaCsrA mutation with respect to the magnitude of the stimulatory effect, perhaps suggesting that CsrA might bind other regions in the promoter? What would the effect be of a double deletion? Also, a limited study evaluating effects of smaller UTR deletion mutations on gene expression could shed more light into the mechanism by which CsrA blocks gene expression. (At this point, there is very little experimentation exploring CsrA mechanism). Minimally, it would be straightforward to map the CsrA binding sit on the UTR RNA (and DNA if it binds DNA) using EMSAs.

**As we mention above, we have now included the results of experiments that point to the involvement of one of the GGA motifs (at position +141 of the mRNA transcript) in conferring inhibitory activity to the 5'UTR_B7_ (see Supplementary Figure 3_)_. The difference between the *Δ5'*UTR_B7_ and *Δ*csrA phenotype with respect to increased virB11-msfGFP reporter expression levels has to take into account the pleiotropic effect a *ΔcsrA* genotype has on the physiology of the bacterial cell (this point is made in the second paragraph of the Results section). Most importantly, the relatively small size of the Δcsr cells can itself lead to an increase in fluorescence levels. Please note that the expression levels of a double mutant are presented in Figure 1D and shows that deleting csrA leads to only a subtle increase in expression levels in the delta-5'UTR_B7_ background. But again, we clearly want to stress that this subtle increase has to be considered in light of the pleiotropic effects of *ΔcsrA* genotype on bacterial physiology. Finally, the experiments represented in Figure 1C and the new experiments in Supplementary Figure 3 clearly disconnects the 5’UTR_B7_ from other regulatory features that might exist upstream or downstream of the 5’UTR in the virB operon: specifically, by studying the behaviour of the 5’UTR_B7_ far away from its natural genetic surroundings (inserted in the distant *amy* locus) and placing it under a constitutive promoter directly followed by *msfGFP* and *gus* reporter genes, respectively.**

4. Is it possible to quantitate CsrA production levels under different environmental/growth conditions – this could provide valuable insights into whether CsrA is acting directly or indirectly to regulate T4SS production.

**The reviewer raises a valid question, and this would indeed be a very valuable experiment. However, at this stage we lack the necessary tools to conduct such an experiment. Coupling msfGFP or any other fluorescent probe directly to CsrA would very likely hamper its effect (CsrA is a small protein and relies on fast and subtle interaction between RNA molecules). We are therefore not able to conduct an analysis like the VirB11 msfGFP analysis conducted here and link these directly to native CsrA levels. This would require accurate single cell proteomics and as far as we are aware these kind of experiments on Bacteria are still far beyond our reach.**

**Having said this, concerning CsrA production levels, even if we were to be able to assess CsrA concentration at the single cell level, regulation when it comes to CsrA is in most cases due to the binding of small RNAs (e.g. CsrB and CsrC in E. coli) to CsrA which govern CsrA’s ability to bind other RNA structures and as such its final “activity”. Therefore, it is not even clear whether CsrA-dependent phenomena are in fact regulated by varying the total cell concentration of CsrA.**

5. Is virD4 subject to CsrA control? This seems to be an obvious question of importance to answer – in the absence of VirD4, the T4SS is presumably nonfunctional, implying there should be coordinate control over gene expression. Simple to address.

**Figure 2A of the revised manuscript presents quantitative real-time polymerase chain reaction (RT-qPCR) of representative genes within the *X. citri* X-T4SS virD and virB operons. The result confirms that VirD4 is in fact under CsrA control. This result is consistent with data previously published by Andrade et al. (2014). Since this study focused on the virB operon, we will refrain from providing any extra information surrounding the *virD* operon which is being currently investigated by our group.**

6. Pg. 9. L. 257. The nutrient limitation studies: what’s the broad conclusion, that there aren’t major changes in gene expression with changes in the nutrients/pH’s tested? This is a pretty limited outcome – corresponding tests with the DeltaCsrA and DeltaUTR variants would provide more insights into the role, if any, of CsrA on maintaining T4SS production levels under these different growth conditions.

**These results are necessary to understand the significance of virB expression and CsrA regulation in *X. citri*. Unlike other large secretion systems that are turned ON-OFF depending on certain conditions (see Introduction, first paragraph), some of which are even under control of CsrA (see Discussion, third paragraph), we show here that, whilst constantly being repressed by CsrA, the T4SS of X. citri is constantly expressed at a basal level (Figure 4D) sufficient to allow it to kill neighboring bacterial cells at a similar level despite significant variations of the tested nutrient conditions. Deleting CsrA or the 5’UTR does lead to higher amounts of T4SS (Figure 3A) and higher killing efficiencies (Figure 3B). This situation, however, is not physiologically relevant since a CsrA null mutant (having no CsrA at all) is much less viable under lab conditions (and most probably in the wild) due to the important roles of CsrA in many physiological processes.**

7. Fig. 5. I really think different fluorescent tags are needed for these studies. Regardless of possible differences in the WT vs DeltaUTR in T4SS focal production, it’s difficult to believe that the two strains can be distinguished and sorted. This would be trivial with GFP and mRFP tags for example. It is also unknown whether mixing of strains under these different growth conditions may affect the overall morphologies and T4SS distributions in one or the other strain, further complicating efforts to distinguish the cells by sorting using a single fluorescent marker.

**As we mentioned in our response to major comment #1 above: " The strength and ingenuity of this experiment is that by using equivalent markers in the two cell lines, the genetic differences are restricted to the presence or absence of the 5'UTRB7. The ability to distinguish the two populations is based on their differences in msfGFP expression levels by using single-cell techniques, which dispense the need for different markers and different strain constructions that could lead to unexpected effects on cell viability and fitness. Please note that these analyses are NOT based on T4SS focal production (like the reviewer seems to have misunderstood in the minor comment sections) but rather on the total amount of msfGFP (cytoplasmic msfGFP) that is produced due to the insertion (not fusion) of the msfgfp genes between the virB11 and virB1 ORFs in the virB opero (strain virB11-msfGFP). In order to clarify this, we have added the fluorescence intensity histograms in Figure 5A that show that the populations are found in two well-separated Gaussian curves at all time points. Furthermore, we point out that the fluorescence intensity cut-off value to distinguish the two populations in the mixture was chosen to err on the side of the less abundant (delta5'UTR) strain. Therefore, the differences in cell numbers shown in Figure 5B and 5C are most likely underestimated."**

8. P. 13. L. 397. It is never shown that CsrA exerts the same repressing power over a wide range of growth conditions, especially since CsrA levels were never shown over the tested conditions. There may well be other regulators that kick in to govern T4SS production under different growth conditions, yielding a more complex picture of regulation than is currently envisioned.

**We do agree with the reviewer that this regulation is course very complex and most probably multi-layered involving an interplay in nucleotide availability, amino acid availability, promoter strength, sigma factors, polymerases, ribosomes and the very complex regulatory pathways leading to CsrA expression and even more importantly its activity governed by regulatory RNAs. However, we respectfully disagree that we did not show that CsrA exerts the same repressing power over a wide range of growth conditions. Please refer to Figure 4A where under different growth conditions CsrA represses virB levels on average 3.8-fold (± 0.4). In this manuscript, we focused on studying and describing the production of a large protein complex that is clearly regulated by CsrA, and show that CsrA regulation is not fully repressive for T4SS in X. citri and does not release its repression under certain conditions (unlike what is commonly observed for other systems). Rather, CsrA keeps a constant but incomplete repression on the virB operon leading to a sustained density of T4SSs in the cell envelope under a wide variety of nutrient conditions**

9. P. 13. L. 402. It would be fairly straightforward to add some more insights about CsrA mechanism of action by testing for DNA binding, or binding to subregions of the UTR, monitoring CsrA levels in vivo to provide a direct test of the assumption that levels don’t change under different conditions, etc.

**We have responded to these issues in our responses above major comments 1, 2 and 3 and minor comments 4 and 8.**

10. The figure legends should only convey information that enables the reader to interpret the figure, not describe the findings or provide conclusions - this is done in the Results and Discussion sections. Several of the figure legends should be revised to delete this redundant material.

**We have stream-lined the figure legends as requested.**

Reviewer #2: N/A

Reviewer #3: Results seems to contain discussion to a limited extent.

**We kindly refer to the response given to the similar question in Part 1: We believe that in some cases it is more adequate to add a phrase or two to explain a result or comment as to its relevance with respect to the recent literature instead of recapitulating the result n the Discussion section in order to make the same point.**
